# Mean Squared Error Representative Points of Pareto Distributions and Their Estimation

**DOI:** 10.3390/e27030249

**Published:** 2025-02-27

**Authors:** Xinyang Li, Xiaoling Peng

**Affiliations:** 1 Faculty of Science and Technology, BNU-HKBU United International College, Zhuhai 519087, China; xinyli@uic.edu.cn; 2 Guangdong Provincial/Zhuhai Key Laboratory of IRADS, BNU-HKBU United International College, Zhuhai 519087, China

**Keywords:** Pareto distributions, MSE-RPs (mean squared error representative points), information gain-based truncation

## Abstract

Pareto distributions are widely applied in various fields, such as economics, finance, and environmental studies. The modeling of real-world data has created a demand for the discretization of Pareto distributions. In this paper, we propose using mean squared error representative points (MSE-RPs) as the discrete representation of Pareto distributions. We demonstrate the uniqueness and existence of these representative points under certain parameter settings and provide a theoretical k-means algorithm for the computation of MSE-RPs for Pareto I and Pareto II distributions. Furthermore, to enhance the applicability of MSE-RPs, we employ three methodological approaches to estimate the MSE-RPs of Pareto distributions. By analyzing the estimation bias under different parameters and methods, we recommend estimating the distribution parameters first before estimating the MSE-RPS for Pareto I and Pareto II distributions. For Pareto III and Pareto IV distributions, we suggest using the $B_{q}$ quantiles for MSE-RP estimation. Building on this, we analyze the sources of estimation bias and propose an effective method for determining the number of MSE-RPs based on information gain truncation. Through simulations and real data studies, we demonstrate that the proposed methods for MSE-RP estimation are effective and can be used to fit the empirical distribution function of data accurately.

## 1. Introduction

The Pareto distribution was originally introduced by the Italian economist Vilfredo Pareto in his seminal work on economics, where it was used as a model for income distribution [1]. Pareto observed that a small proportion of the population tends to control the majority of wealth—a phenomenon commonly referred to as the “80/20 rule”, meaning that 20% of people own 80% of the income. Since then, this heavy-tailed distribution has been widely applied in fields such as economics, finance, and risk management [2], particularly for modeling phenomena characterized by significant inequality or extreme outcomes. Examples include modeling stock return distributions [3] and the extreme tails of financial and insurance loss datasets [4]. The classical Pareto distribution, often referred to as the Pareto Type I distribution, is defined by a skewed heavy-tailed model:

(1) $$F_{X}\left(x\right)=1-{\left(\frac{x}{\sigma }\right)}^{-\alpha },x\geq \sigma >0,\alpha >0,$$

where σ>0 is the scale parameter, typically representing the minimum income in income models, and α>0 is the shape parameter, commonly referred to as the Pareto index. The Pareto index α serves as a measure of inequality, with larger values indicating more equitable distributions. For income analysis, the Pareto index typically fluctuates around 1.5 [5].

While the Pareto Type I distribution performs well in modeling the income of high-income groups, it often fails to capture income distributions across the entire population. To address this limitation, economists and statisticians have extended the Pareto distribution by introducing additional flexibility through the location parameter μ and inequality parameter γ, resulting in the development of Pareto Type II, III, and IV distributions [6]. These generalized Pareto distributions have found broader applications. For instance, the Pareto Type II distribution is used for flood modeling and rainfall analysis [7], the Pareto Type III distribution is commonly applied to earthquake intensity modeling [8], and the Pareto Type IV distribution is employed in insurance risk analysis [9].

In statistical research, most studies on the Pareto family focus on data modeling and statistical inference. A major challenge in statistical inference lies in the complex expressions of the generalized Pareto family. Parameter estimation for the Pareto family often involves significant computational effort due to the lack of closed-form expressions for methods such as moment estimation (ME) and maximum likelihood estimation (MLE). Additionally, there has been a lot of research focus on the performance of parameter estimation in small samples and the robustness of these estimates [10,11,12]. In practical data modeling, it is often necessary to discretize continuous data into discrete models while retaining the desirable properties of continuous distributions. For example, Ghosh [13] proposed a discrete version of the Pareto IV distribution derived from rounding continuous random variables to improve the accuracy of lifetime modeling.

In this paper, we focus on the discretization of the Pareto family under the minimum mean squared error (MSE). In statistics, for a given continuous distribution, we can identify optimal discrete approximations, referred to as representative points (RPs), based on different kinds of errors. Different error metrics yield different types of RPs, such as mean squared error representative points (MSE-RPs) [14], Monte Carlo representative points (MC-RPs) [15], and quasi-Monte Carlo representative points (QMC-RPs) [16]. Among these, MSE-RPs are the most widely used, as they demonstrate superior performance over other RPs in distribution fitting [15].

MSE-RPs were initially applied to optimize signal transmission [17]. Since signal distortion in transmission is defined as the expected squared error between the quantizer input and output, which aligns with the MSE-RP criterion, Max successfully applied it to minimize signal distortion using quantizers with fixed output levels [18]. Fang and He [14] further advanced the computation and applications of MSE-RPs. Subsequently, MSE-RPs have been widely studied and applied in various fields, including clothing standardization [14], determining optimal sizes and shapes for protective masks [19], signal and image processing, information theory [20], psychiatric classification [21], statistical inference using resampling techniques [22], and reducing variance in one-dimensional Monte Carlo estimations [23]. They have also been utilized in parameter estimation, such as using MSE-RPs from the gamma distribution as standard samples to improve parameter estimation accuracy [24].

The majority of existing studies on MSE-RPs have focused on univariate continuous distributions, such as normal, exponential, gamma, Weibull, logistic, and mixed normal distributions. However, no studies have been conducted on MSE-RPs derived from Pareto distributions. Owing to the complexity of Pareto density functions, the study of MSE-RP generation and estimation presents significant challenges. Current studies propose two main approaches to generate MSE-RPs for continuous distributions. The first approach relies on the self-consistency property shared by MSE-RPs and k-means cluster centers, which has led to the development of k-means-based algorithms for generating MSE-RPs. This method was initially proposed by Lloyd [25] and Max [18], and is referred to as the Lloyd I algorithm, specifically designed for univariate continuous distributions. To mitigate the impact of initial values and training sets on MSE-RPs, Linde et al. [26] proposed an iterative algorithm, referred to as the LBG algorithm. Fang et al. [27] later developed an enhanced version of the LBG algorithm, employing number-theoretic methods to generate initial points and training sets, which is referred to as the NTLBG algorithm. The second approach derives from the definition of MSE-RPs, with the objective of finding a discrete distribution that minimizes the MSE between it and the density function. This involves solving a series of equations for obtaining the MSE-RPs, a method referred to as the Fang–He algorithm [14]. Compared to k-means based methods, the Fang–He algorithm offers greater accuracy in the computation of MSE-RPs. However, it requires verifying the convergence of the algorithm, which needs to be addressed separately for each specific distribution.

In practical applications, it is often necessary to optimally discretize continuous data, which involves estimating the MSE-RPs of the underlying continuous distribution that the data follow. Tarpey [28] proposed four methods for estimating the MSE-RPs of univariate continuous distributions: maximum likelihood estimation, semi-parametric estimation, quantile estimation, and the k-means algorithm. Subsequent research has expanded these methods. For instance, Matsuura et al. [29] developed an optimal estimation approach for the t-distribution and extended it to the location–scale family and multivariate distributions. In the case of the Pareto distribution, we primarily study the latter two methods for estimating MSE-RPs.

The Pareto distribution is a right-skewed distribution, where the estimation bias of the quantiles at the tail is relatively large. Consequently, when using the quantile method to estimate the MSE-RPs located in the tail, the bias will also be significant. Similarly, due to the smaller number of samples in the tail, the k-means method also introduces substantial bias. The inaccurate estimation of MSE-RPs in the tail increases the bias of the overall estimate. In fact, the amount of information contained in the MSE-RPs at the tail is very limited. This information can be quantified using information gain (IG), which has been applied to evaluate the information content of MSE-RPs in mixed normal distributions [30]. Based on this, we introduce a truncation method based on IG to determine the sufficient number of MSE-RPs. This approach aims to reduce the estimation bias while preserving as much information as possible in the MSE-RPs.

In conclusion, our study focus on the generation and estimation of MSE-RPs for the four types of Pareto distributions. We examine the properties of MSE-RPs for Pareto distributions and propose a reliable algorithm for their generation based on the properties. In addition, simulation results reveal that existing methods for estimating MSE-RPs suffer from significant bias when applied to skewed and heavy-tailed distributions. To address this issue, we introduce the innovative concept of IG-truncated representative points. This approach also provides a new perspective for estimating MSE-RPs for other heavy-tailed distributions.

The structure of this paper is as follows: Section 2 introduces the fundamentals of the Pareto family. Section 3 presents the MSE-RPs of Pareto distributions, including their properties, computation methods, and results. Section 4 compares different methods for estimating the MSE-RPs of Pareto distributions and proposes an IG-based truncation method for selecting the number of MSE-RPs. In Section 5, a real dataset is analyzed in order to illustrate the proposed methods. Finally, this paper is concluded in Section 6.

## 2. Preliminaries of Pareto Distributions

### 2.1. Four Types of Pareto Distributions

The two-parameter Pareto distribution was first introduced by Vilfredo Pareto, and its cumulative distribution function (CDF) is given in Equation (1). This distribution is also known as the Pareto Type I distribution, denoted as X∼P(I)(σ,α).

Building upon this, Pareto proposed two additional variations. The Pareto Type II distribution, also known as the Lomax distribution, is a three-parameter distribution derived from the analysis of business failure data [31]. Its CDF is given by

(2) $$F_{X}\left(x\right)=1-{\left(1+\frac{x-\mu }{\sigma }\right)}^{-\alpha },x\geq \mu ,\sigma ,\alpha >0,$$

where μ is the location parameter, generally assumed to be non-negative. Additionally, α>1 is required to ensure a finite expected value. This distribution is denoted as P(II)(μ,σ,α).

The Pareto Type III distribution is another three-parameter distribution, and its CDF is defined as follows:

(3) $$F_{X}\left(x\right)=1-{\left(1+{\left(\frac{x-\mu }{\sigma }\right)}^{\frac{1}{\gamma }}\right)}^{-1},x>\mu ,\sigma ,\gamma >0,$$

denoted as P(III)(μ,σ,γ). When μ=0 and γ≤1, the parameter γ represents the Gini index for inequality.

In 1983, Arnold introduced the Pareto Type IV distribution, also referred to as the Burr distribution. Its CDF is expressed as follows:

(4) $$F_{X}\left(x\right)=1-{\left(1+{\left(\frac{x-\mu }{\sigma }\right)}^{\frac{1}{\gamma }}\right)}^{-\alpha },x\geq \mu ,\sigma ,\gamma ,\alpha >0,$$

denoted as Pareto(IV)(μ,σ,γ,α).

The four types of Pareto distributions are not entirely independent and can be transformed into one another by adjusting the parameters. Specifically, Pareto(I), Pareto(II), and Pareto(III) distributions are special cases of the Pareto(IV) distribution. These relationships are expressed as follows:

(5) $$\begin{array}{ccc} P(I)(\sigma ,\alpha ) & = & P(IV)(\sigma ,\sigma ,1,\alpha ),\\ P(II)(\mu ,\sigma ,\alpha ) & = & P(IV)(\mu ,\sigma ,1,\alpha ),\\ P(III)(\mu ,\sigma ,\gamma ) & = & P(IV)(\mu ,\sigma ,\gamma ,1). \end{array}$$

By modifying the parameters of the Pareto IV distribution, the other three types can be derived. Thus, Pareto I, II, and III can all be viewed as special cases of the Pareto IV distribution.

In its early development, the Pareto distribution was considered a specific form of the exponential distribution, as it can be transformed into an exponential distribution. If U∼Γ(1,1) is a standard exponential random variable, then $X=\sigma e^{\frac{U}{\alpha }}∼P\left(I\right)\left(\sigma ,\alpha \right)$ and $Y=\mu +\sigma \left(e^{\frac{U}{\alpha }}-1\right)∼P\left(II\right)\left(\mu ,\sigma ,\alpha \right)$.

Furthermore, all four types of Pareto distributions can be transformed into their standard forms by adjusting their parameters. For k>0 and γ>0, the following hold:

- If X∼P(I)(1,α), then
  (6) $$Z=\sigma X^{1/k}∼P\left(I\right)\left(\sigma ,k\alpha \right).$$
- If X∼P(II)(0,1,α), then
  (7) $$Z=\mu -\sigma +\sigma {\left(1+X\right)}^{1/k}∼P\left(II\right)\left(\mu ,\sigma ,k\alpha \right).$$
- If X∼P(III)(0,1,1), then
  (8) $$Z=\mu +\sigma X^{\gamma }∼P\left(III\right)\left(\mu ,\sigma ,\gamma \right).$$
- If X∼P(IV)(0,1,1,α), then
  (9) $$Z=\mu +\sigma {\left[{\left(1+X\right)}^{1/k}-1\right]}^{\gamma }∼P\left(IV\right)\left(\mu ,\sigma ,\gamma ,k\alpha \right).$$

### 2.2. Parameter Estimation of Pareto Distributions

Although numerous studies have explored parameter estimation for Pareto distributions, most focus on the Pareto Type I distribution due to its simplicity, widespread use, and interpretability. Here, we divide the discussion into two parts: parameter estimation for the Pareto Type I distribution and for the Pareto Type II–IV distributions.

1. Pareto Type I Distribution:
  (a) Maximum Likelihood Estimation (MLE): Assume a sample $X_{1},\ldots ,X_{n}$ is drawn from P(I)(σ,α), and let $X_{\left(n,1\right)}<\cdots <X_{\left(n,n\right)}$ denote the order statistics. The likelihood function is given by
    (10) $$L\left(\alpha ,\sigma |X_{1},\ldots ,X_{n}\right)=\prod_{i=1}^{n}\frac{\alpha \sigma^{\alpha }}{X_{i}^{\alpha +1}}I\left(\sigma \leq X_{\left(n,1\right)}\right).$$
    When α is known, the likelihood function is a decreasing function of σ. Thus, the MLE of σ is
    (11) $${\hat{\sigma }}_{ML}=X_{\left(n,1\right)}.$$
    Taking the logarithm of the likelihood function and differentiating with respect to α yields
    (12) $${\hat{\alpha }}_{ML}=\frac{n}{\sum_{i=1}^{n}\log\left(X_{i}/{\hat{\sigma }}_{ML}\right)}.$$
    While MLE is asymptotically consistent, normal, and efficient, it is not the minimum variance unbiased estimator (MVUE). The MVUE for this distribution is given by
    (13) $${\hat{\sigma }}^{U}=\left[1-{\left(n-1\right)}^{-1}{\hat{\alpha }}_{ML}^{-1}\right]{\hat{\sigma }}_{ML},\;{\hat{\alpha }}^{U}=\frac{n-2}{n}{\hat{\alpha }}_{ML}.$$
  (b) Moment Estimation (ME): For a Pareto Type I distribution with α>1, the first moment is
    (14) $$E\left(X\right)=\frac{\alpha \sigma }{\alpha -1},\;\alpha >1.$$
    Using the first moment, the method of moments estimators are as follows:
    (15) $${\hat{\alpha }}_{ME}=\frac{n\bar{X}-X_{\left(n,1\right)}}{n(\bar{X}-X_{\left(n,1\right)})},\;{\hat{\sigma }}_{ME}=\left(1-\frac{1}{n{\hat{\alpha }}_{ME}}\right)X_{\left(n,1\right)}.$$
    While ${\hat{\alpha }}_{ME}$ requires α>1, ${\hat{\sigma }}_{ME}$ does not rely on this condition, as it is derived from the CDF of $X_{\left(n,1\right)}$ [10]. Simulations by Lu and Tao [32] indicate that ME performs better than MLE for estimating σ, provided the estimation of σ is independent of α.
2. Pareto Type II–IV Distributions:
  (a) Maximum Likelihood Estimation (MLE): For simplicity, we focus on the Pareto Type IV distribution, as Pareto Types II and III are special cases (see Equation (5)). The likelihood function for a sample $X_{1},\ldots ,X_{n}$ from P(IV)(μ,σ,γ,α) is
    (16) $$\begin{array}{cc} L(\mu ,\sigma ,\gamma ,\alpha |X_{1},\ldots ,X_{n}) & =\left(\frac{1}{\gamma }-1\right)\sum_{i=1}^{n}\log\left(\frac{X_{i}-\mu }{\sigma }\right)\\ & \;-\left(\alpha +1\right)\sum_{i=1}^{n}\log\left(1+{\left(\frac{X_{i}-\mu }{\sigma }\right)}^{1/\gamma }\right)\\ & \;-n\log\gamma -n\log\sigma +n\log\alpha . \end{array}$$
    The order statistic $X_{\left(n,1\right)}$ serves as a consistent estimator of μ. Once μ is estimated, the problem can be reduced to estimating the parameters of P(IV)(0,σ,γ,α) using the remaining n−1 samples, $X_{\left(n,2\right)},\ldots ,X_{\left(n,n\right)}$. Taking partial derivatives of the log-likelihood function with respect to σ, γ, and α yields
    (17) $$\begin{array}{cc} \frac{\partial \log L}{\partial \sigma } & =\frac{\alpha (n-1)}{\gamma \sigma }-\frac{\alpha +1}{\gamma \sigma }\sum_{i=1}^{n-1}\frac{1}{1+{\left(X_{i}/\sigma \right)}^{1/\gamma }}=0,\\ \frac{\partial \log L}{\partial \gamma } & =\frac{\alpha }{\gamma^{2}}\sum_{i=1}^{n-1}\log\left(\frac{X_{i}}{\sigma }\right)-\frac{\alpha +1}{\gamma^{2}}\sum_{i=1}^{n-1}\frac{\log\left(X_{i}/\sigma \right)}{1+{\left(X_{i}/\sigma \right)}^{1/\gamma }}-\frac{n-1}{\gamma }=0,\\ \frac{\partial \log L}{\partial \alpha } & =-\sum_{i=1}^{n-1}\log\left(1+{\left(\frac{X_{i}}{\sigma }\right)}^{1/\gamma }\right)+\frac{n-1}{\alpha }=0. \end{array}$$
    Solving these equations yields the MLEs for P(IV)(0,σ,γ,α).
  (b) Moment Estimation (ME): Direct computation of moments for Pareto distributions is challenging. Arnold proposed a method based on constructing a statistic relying on the Gamma distribution [6]. Define
    (18) $$X=\mu +\sigma {\left(\frac{U_{1}}{U_{2}}\right)}^{\gamma }∼P\left(IV\right)\left(\mu ,\sigma ,\gamma ,\alpha \right),$$
    where $U_{1}∼\Gamma \left(1,1\right)$ and $U_{2}∼\Gamma \left(\alpha ,1\right)$ are independent. Using the moments of the Gamma distribution, the δ-th moment of *X* is
    (19) $$E\left(X^{\delta }\right)=\sigma^{\delta }\frac{\Gamma (1+\delta \gamma )\Gamma (\alpha -\delta \gamma )}{\Gamma (\alpha )},\;-\frac{1}{\gamma }<\delta <\frac{\alpha }{\gamma }.$$
    By constructing equations from these moments and using $X_{\left(n,1\right)}$ as a consistent estimator of μ, the moment estimators for Pareto Type IV distributions can be obtained. However, the existence of moments depends on the parameter conditions.

## 3. MSE-RPs of Pareto Distributions

### 3.1. Definition and Properties of MSE-RPs

Given a continuous random variable X∼F(x), a set of points $\left\{y_{i}\in R^{d},\;i\in \left\{1,\cdots ,m\right\}\right\}$ and their associated probabilities form the MSE-RPs of F(x) if and only if the following condition is satisfied:

(20) $$E\left[d^{2}\left(x|y_{1},\cdots ,y_{m}\right)\right]=\min_{\begin{array}{c} \xi_{i}\in R^{d}\\ i=1,\cdots ,m \end{array}}E\left[d^{2}\left(x|\xi_{1},\cdots ,\xi_{m}\right)\right],$$

where

(21) $$d^{2}\left(x|\xi_{1},\cdots ,\xi_{m}\right)=\min_{\begin{array}{c} j\in \{1,\cdots ,m\} \end{array}}∥x-\xi_{j}∥$$

represents the minimum $\ell_{2}$-norm distance between x and $\xi_{j}$, j∈{1,⋯,m}.

The probability of $y_{i}\in R^{d}$ is given by $P\left(y_{i}\right)=P\left(X|X\in V_{i}\right)$, where

(22) $$V_{i}=\left\{x\in R^{d}|{\left(x-y_{i}\right)}^{\prime }\left(x-y_{i}\right)⩽{\left(x-y_{j}\right)}^{\prime }\left(x-y_{j}\right),\;\forall i\neq j\right\},\;i\in \left\{1,\cdots ,m\right\}.$$

MSE-RPs essentially represent the discretization of a continuous distribution. Through $y_{i}$ and their associated probabilities, the support of the continuous distribution is divided into *m* adjacent and non-overlapping regions, known as Voronoi partitions. For each partition $V_{i}$, its center $y_{i}$ satisfies

(23) $$E\left[X|X\in V_{i}\right]=y_{i},\;i=1,\ldots ,m.$$

These centers are also referred to as self-consistent points. Tarpey and Flury [33] proved that the set of self-consistent points minimizing the MSE is precisely the set of MSE-RPs.

For one-dimensional continuous distributions, MSE-RPs can be arranged as $y_{1}<y_{2}<\cdots <y_{m}$. In this case, the probability corresponding to $y_{i}$ is given by

(24) $$P_{m}\left(y_{i}\right)=\int_{M_{i}}^{M_{i+1}}f\left(x\right)\;dx,\;i=1,\cdots ,m,$$

where $M_{1}=F^{-1}\left(0\right)$, $M_{i}=\frac{y_{i-1}+y_{i}}{2},\;i=2,\cdots ,m$, and $M_{m+1}=F^{-1}\left(1\right)$.

For location–scale distributions with standard forms, it suffices to study their MSE-RPs in the standard case. These standard MSE-RPs can then be extended to the full parameter space using the location–scale transformation properties of MSE-RPs proposed by Zoppè [34]. Since the Pareto Type IV distribution belongs to the location–scale family and Pareto Types I-III are special cases of it, we focus on the MSE-RPs of P(IV)(0,1,γ,α). This leads to the following corollary.

**Corollary** **1.** *If $\left\{y_{i},i=1,\cdots ,m\right\}$ are the m MSE-RPs of P(IV)(0,1,γ,α), then $\left\{z_{i}=\sigma y_{i}+\mu ,i=1,\cdots ,m\right\}$ are the m MSE-RPs of P(IV)(μ,σ,γ,α).*

### 3.2. Existence and Uniqueness

The existence of MSE-RPs for a continuous distribution requires that its first two moments exist [35]. For Pareto distributions, the conditions for the existence of the second moment are as follows:

(25) $$\begin{array}{cc} P(I)(\sigma ,\alpha ): & \;\alpha >2,\\ P(II)(\mu ,\sigma ,\alpha ): & \;\alpha >2,\\ P(III)(\mu ,\sigma ,\gamma ): & \;0<\gamma <\frac{1}{2},\\ P(IV)(\mu ,\sigma ,\gamma ,\alpha ): & \;\frac{\alpha }{\gamma }>2. \end{array}$$

If the parameters of a Pareto distribution do not satisfy the above conditions, its MSE-RPs do not exist.

Next, we examine the shape of the density function. According to the theorem by Trushkin [36], if the density function f(x) of a continuous distribution is unimodal and log-concave, the MSE-RPs of the distribution are unique. The density function of P(IV)(0,1,γ,α) is unimodal, and we provide the following lemma regarding its shape.

**Lemma** **1.** *Let x∼P(IV)(0,1,γ,α), with $\frac{1}{\gamma }>1$. Define a random variable $z=\frac{x^{1/\gamma }}{1+x^{1/\gamma }},0<z<1$. Then, there exists a $z_{2}^{*}$ given by*

(26) $$z_{2}^{*}=\frac{\left(\alpha +1\right)\left(\frac{1}{\gamma }-1\right)+\sqrt{{\left(\alpha +1\right)}^{2}{\left(\frac{1}{\gamma }-1\right)}^{2}+4\left(\alpha +1\right)\left(\frac{1}{\gamma }-1\right)}}{2(\alpha +1)/\gamma },$$

*such that when $z\in (0,z_{2}^{*})$, the density function of P(IV)(0,1,γ,α) is log-concave. Moreover, as α→+∞, $z_{2}^{*}\to 1-\gamma$.*

We prove that the density function is log-concave by demonstrating that its logarithmic first derivative is a strictly decreasing function. The detailed proof is provided in Appendix A. Using this theorem, we establish the existence and uniqueness of the MSE-RPs for the Pareto IV distribution under the condition $\frac{1}{\gamma }>1$.

For other parameter settings where the density function is not log-concave, we rely on proving the uniqueness of the solutions to the system of equations to demonstrate the uniqueness of the MSE-RPs. This approach was proposed by Fang and He [14] in their proof of the uniqueness of MSE-RPs for the normal distribution. The method involves showing that the equations derived from setting the first derivative of (20) to zero have a unique solution.

By taking the partial derivative of (20), we obtain

(27) $$\frac{\partial E[d^{2}\left(x|y_{1},\cdots ,y_{m}\right)]}{\partial y_{i}}=0,\;i=1,\cdots ,m,$$

which simplifies to the following system of equations:

(28) $$L_{i}=\int_{M_{i}}^{M_{i+1}}\left(x-y_{i}\right)f\left(x\right)\;dx=0,\;i=1,\cdots ,m.$$

For i=1,⋯,m−1, taking the partial derivative of $L_{i}$ with respect to $y_{i+1}$ gives

(29) $$\frac{\partial L_{i}}{\partial y_{i+1}}=\frac{1}{2}f\left(M_{i+1}\right)\left(M_{i+1}-y_{i}\right)>0,\;i=1,\cdots ,m-1.$$

Similarly, for $L_{m}$, taking the partial derivative with respect to $y_{m}$ yields

(30) $$\frac{\partial L_{m}}{\partial y_{m}}=\frac{1}{2}f\left(M_{i}\right)\left(-M_{m}+y_{m}\right)<0.$$

From (29) and (30), it can be seen that for the *i*-th equation, given that $y_{j,j<i+1}$ are known, the equation has a unique solution for $y_{i+1}$. When solving the system of equations, an initial value is typically assigned to $y_{1}$, and the values are sequentially substituted into the equations to solve for subsequent variables. For m−1 unknowns, there are *m* equations, with the last two equations both involving $y_{m}$. By iteratively comparing the solutions of the last two equations for $y_{m}$, the system converges to a unique solution when the two solutions are equal.

Next, we analyze the existence conditions for solutions to the system of Equations (29) and (30) for P(IV)(0,1,γ,α). It is known that $L_{i}$ is a monotonic function of $y_{i+1}$, where $y_{i+1}>0$. The condition for the existence of a solution to $L_{i}=0$ is

(31) $$L_{i}\left(y_{i+1}\to y_{i}\right)*L_{i}\left(y_{i+1}\to +\infty \right)<0.$$

**Theorem** **1.** *For the Pareto distribution P(IV)(0,1,γ,α), the system of Equation (29) has a unique solution when $y_{1}<E\left(X\right)$ and $\left\{y_{i}<\frac{E\left(X\right)-\sum_{j=1}^{i-1}y_{j}}{1-F(M_{i})},i=2,\cdots ,m-1\right\}$.*

The proof is provided in Appendix A.

### 3.3. Generation of MSE-RPs

From the definition of MSE-RPs, it can be seen that solving for MSE-RPs is essentially an optimization problem concerning (20). The conditions for the existence of a solution to this optimization problem are strict, and it may not be possible to derive explicit solutions for MSE-RPs by taking partial derivatives of the objective function. To address this issue, Fang and He [14] proposed an iterative method for solving a system of nonlinear Equation (28). Alternatively, MSE-RPs can also be obtained by leveraging their property as a set of self-consistent points that minimize the mean squared error (MSE). Since the centroids of the k-means algorithm are also self-consistent points, when MSE-RPs exist and are unique, k-means can be used to find the centroids, with the set of centroids that minimizes the MSE being the MSE-RPs. This method is efficient and fast but requires multiple iterations until the results stabilize and may converge to a local minimum.

To address the drawbacks of these methods, Xu et al. [37] proposed a k-means method based on the special properties of MSE-RPs for exponential distributions. This method avoids solving large systems of nonlinear equations and prevents k-means from converging to local minima. Inspired by this, we propose a similar method for generating MSE-RPs for Pareto distributions.

For Pareto I and II distributions, substituting their density functions into the last equation in (28) and simplifying yields

(32) $$\begin{array}{cc} P(I)(1,\alpha ): & \frac{\alpha }{-\alpha +1}x^{-\alpha +1}+y_{m}x^{-\alpha }{|}_{(y_{m-1}+y_{m})/2}^{+\infty }=0.\\ P(II)(0,1,\alpha ): & \frac{\alpha }{-\alpha +1}{\left(1+x\right)}^{-\alpha +1}+\left(1+y_{m}\right){\left(1+x\right)}^{-\alpha }{|}_{(y_{m-1}+y_{m})/2}^{+\infty }=0. \end{array}$$

Simplifying further, we obtain

(33) $$\begin{array}{cc} P(I)(1,\alpha ): & \alpha y_{m-1}+\left(2-\alpha \right)y_{m}=0.\\ P(II)(0,1,\alpha ): & \alpha y_{m-1}+2+\left(2-\alpha \right)y_{m}=0. \end{array}$$

These equations describe the relationship between the last two points in a set of MSE-RPs for Pareto I and II distributions, leading to the following theorems.

**Theorem** **2.** *Suppose $y_{1}<y_{2}<\ldots <y_{m}$ are the m MSE-RPs of a Pareto distribution P(I)(1,α|α>2). Then, the following relationship holds for $y_{m-1}$ and $y_{m}$:*

(34) $$y_{m}=\frac{\alpha y_{m-1}}{\alpha -2}.$$

**Theorem** **3.** *Suppose $y_{1}<y_{2}<\ldots <y_{m}$ are the m MSE-RPs of a Pareto distribution P(II)(0,1,α|α>2). Then, the following relationship holds for $y_{m-1}$ and $y_{m}$:*

(35) $$y_{m}=\frac{\alpha y_{m-1}+2}{\alpha -2}.$$

Unfortunately, for Pareto III and IV distributions, no similar theorems exist. Therefore, we propose the following Algorithm 1 for Pareto I and II distributions. 
**Algorithm 1:** Theoretical k-means algorithm for Pareto I and II distributionsStep 1: For a given pdf p(x), the number of MSE-RPs *m*, initial iteration t=0, and tolerance ϵ, input an initial set of number-theoretic methods representative points (NTM-RPs) [16] $y_{1}^{\left(t\right)}<y_{2}^{\left(t\right)}<\ldots <y_{m}^{\left(t\right)}$. Define a partition of R as:$$I_{i}^{\left(t\right)}=\left(a_{i}^{\left(t\right)},a_{i+1}^{\left(t\right)}\right],\;i=1,\ldots ,m-1,\;I_{m}^{\left(t\right)}=\left(a_{m-1}^{\left(t\right)},a_{m}^{\left(t\right)}\right),$$
where$$a_{1}^{\left(t\right)}=-\infty ,\;a_{i}^{\left(t\right)}=\frac{y_{i-1}^{\left(t\right)}+y_{i}^{\left(t\right)}}{2},\;i=2,\ldots ,m,\;a_{m}^{\left(t\right)}=\infty .$$Step 2: Calculate probabilities:$$p_{j}^{\left(t\right)}=\int_{I_{j}^{\left(t\right)}}p\left(x\right)\;x,\;j=1,\ldots ,m;$$Step 3: Calculate conditional means:For j=1,⋯,m−1:$$y_{j}^{\left(t+1\right)}=\frac{\int_{I_{j}^{\left(t\right)}}xp\left(x\right)\;dx}{\int_{I_{j}^{\left(t\right)}}p\left(x\right)\;dx}=\frac{\int_{I_{j}^{\left(t\right)}}xp\left(x\right)\;dx}{p_{j}^{\left(t\right)}},\;j=1,\ldots ,m-1.$$For P(I)(1,α):$$y_{m}^{\left(t+1\right)}=\frac{\alpha }{\alpha -2}y_{m-1}^{\left(t+1\right)}.$$For P(II)(0,1,α):$$y_{m}^{\left(t+1\right)}=\frac{\alpha y_{m-1}^{\left(t+1\right)}+2}{\alpha -2}.$$Step 4: Sort $\left\{y_{1}^{\left(t+1\right)},\ldots ,y_{m}^{\left(t+1\right)}\right\}$ from smallest to largest.Step 5: Calculate $|y_{m}^{\left(t+1\right)}-b|$, where$$b=\frac{\int_{I_{m}^{\left(t\right)}}xp\left(x\right)\;dx}{p_{m}^{\left(t\right)}}.$$Step 6: If $|y_{m}^{\left(t+1\right)}-b|<\epsilon$, the process stops, and $\left\{y_{j}^{\left(t\right)}\right\}$ are delivered as the MSE-RPs of the distribution with probabilities $\left\{p_{j}^{\left(t\right)}\right\}$. Otherwise, let t:=t+1 and return to Step 1.

This method not only guarantees that the points obtained are MSE-RPs by leveraging Theorems 2 and 3, but also significantly improves convergence speed by replacing the computation of $y_{m}$.

For Pareto III and IV distributions, solving (28) directly is challenging due to the presence of terms such as $x^{1/\gamma },x^{2/\gamma },\ldots ,x^{(\gamma -1)/\gamma }$ and $\ln(1+x^{1/\gamma })$, which make the equations complex and computationally expensive. Thus, we also adopt the k-means method (Algorithm 2) for obtaining MSE-RPs for these distributions [38].  
**Algorithm 2:** Parametric k-means algorithmStep 1: For a given pdf p(x), the number of MSE-RPs *m*, and initial iteration t=0, input an initial set of points $y_{1}^{\left(t\right)}<y_{2}^{\left(t\right)}<\ldots <y_{m}^{\left(t\right)}$. Define a partition of R as:$$I_{i}^{\left(t\right)}=\left(a_{i}^{\left(t\right)},a_{i+1}^{\left(t\right)}\right],\;i=1,\ldots ,m-1,\;I_{m}^{\left(t\right)}=\left(a_{m-1}^{\left(t\right)},a_{m}^{\left(t\right)}\right),$$
where:$$a_{1}^{\left(t\right)}=-\infty ,\;a_{i}^{\left(t\right)}=\frac{y_{i-1}^{\left(t\right)}+y_{i}^{\left(t\right)}}{2},\;i=2,\ldots ,m,\;a_{m}^{\left(t\right)}=\infty .$$Step 2: Calculate probabilities:$$p_{j}^{\left(t\right)}=\int_{I_{j}^{\left(t\right)}}p\left(x\right)dx,\;j=1,\ldots ,m;$$Step 3: Calculate conditional means:$$y_{j}^{\left(t+1\right)}=\frac{\int_{I_{j}^{\left(t\right)}}xp\left(x\right)dx}{\int_{I_{j}^{\left(t\right)}}p\left(x\right)dx}=\frac{\int_{I_{j}^{\left(t\right)}}xp\left(x\right)dx}{p_{j}^{\left(t\right)}},j=1,\cdots ,m.$$Step 4: If $\left\{y_{j}^{\left(t\right)}\right\}$ and $\left\{y_{j}^{\left(t+1\right)}\right\}$ are identical, the process stops, and $\left\{y_{j}^{\left(t\right)}\right\}$ are delivered as the MSE-RPs of the distribution with probabilities $\left\{p_{j}^{\left(t\right)}\right\}$. Otherwise, let t:=t+1 and return to Step 1.

Using the parametric k-means method is highly effective for calculating MSE-RPs. However, this method cannot guarantee that the output is always the MSE-RPs and may instead converge to other sets of self-consistent points, as pointed out by Stampfer and Stadlober [38]. Therefore, we maximize the sample size for each calculation and select the set of points with the smallest MSE until the results stabilize. Since calculating MSE-RPs for Pareto III and IV distributions is slow, we provide up to 31 MSE-RPs and their corresponding probabilities for reference in Appendix B.

## 4. Estimation of MSE-RPs from Pareto Distributions

Currently, methods for estimating MSE-RPs from a sample can be categorized into three types based on whether the underlying distribution type and parameters are known:

1. The first type requires knowledge of the distribution type. After estimating the distribution parameters, the MSE-RPs are computed directly from the estimated distribution.
2. The second type also requires knowledge of the distribution type but does not involve parameter estimation. Instead, it estimates MSE-RPs by locating the sample quantiles corresponding to the positions of MSE-RPs. This method is limited to distributions with location–scale properties.
3. The third type does not require any prior information about the distribution. It directly estimates MSE-RPs from the sample by leveraging the property that k-means cluster centers are also self-consistent points. MSE-RPs are estimated as the centers of clusters formed by classifying the sample.

For the first type of method, estimating MSE-RPs for Pareto distributions, faces challenges regardless of whether the method of ME or MLE is used. The ME method requires the existence of moments, while the MLE method is computationally complex, making it difficult to achieve accurate results. Therefore, in the simulations presented in this paper, we estimate MSE-RPs under the assumption that the parameters γ and α are known, and only μ and σ are estimated. Specifically, our estimates are as follows:

- For a sample following $P\left(I\right)\left(\hat{\sigma },\alpha \right)$, the MSE-RPs are estimated as
  $$\hat{y}=\hat{\sigma }y,$$
  where y are the MSE-RPs of P(I)(1,α).
- For a sample following $P\left(IV\right)\left(\hat{\mu },\hat{\sigma },\gamma ,\alpha \right)$, the MSE-RPs are estimated as
  $$\hat{y}=\hat{\sigma }y+\hat{\mu },$$
  where y are the MSE-RPs of P(IV)(0,1,γ,α).

The second type of method views MSE-RPs as specific quantiles of the distribution. For distributions with location–scale properties, the positions of quantiles remain unchanged under parameter transformation. Thus, the MSE-RPs can be estimated by determining their corresponding quantile positions in the standard distribution and then estimating these quantiles in the sample. The steps are as follows:

1. Compute the MSE-RPs $y_{1},\cdots ,y_{m}$ of the continuous distribution F(x).
2. Compute the positions $q_{i}$ of $y_{i}$ in the standard distribution F(x):
  $$q_{i}=\int_{-\infty }^{y_{i}}f\left(x\right)\;dx,\;i=1,\cdots ,m.$$
3. In an independent and identically distributed sample $S_{1},\ldots ,S_{n}$, estimate the sample quantiles $Q(q_{i})$ for i=1,⋯,m, which serve as the estimated MSE-RPs for the sample.

The accuracy of this method depends heavily on the choice of the quantile estimation method. Different distributions have unique characteristics, and the optimal method for quantile estimation may vary. Since the Pareto distribution is a heavy-tailed distribution, we consider the following four quantile estimation methods, which are known to perform well in the tails of distributions. Consider estimating the quantile Q(q); the sample size is *n* and X(i) is the *i*-th order statistic of the sample:

- $HD_{q}$ quantile estimator [39]:
  (36) $$HD_{q}=\sum_{i=1}^{n}\left[\int_{i-1/n}^{i/n}\frac{\Gamma (n+1)}{\Gamma ((n+1)q)\Gamma ((n+1)p)}t^{(n+1)q-1}{\left(1-t\right)}^{(n+1)p-1}\;dt\right]X_{\left(i\right)},\;p=1-q.$$
- $B_{q}$ quantile estimator [40]:
  (37) $$B_{q}=\sum_{i=1}^{n}\left[n^{-1}\cdot \frac{\Gamma (n+1)}{\Gamma (i)\Gamma (n-i+1)}q^{i-1}{\left(1-q\right)}^{n-i}\right]X_{\left(i\right)}.$$
- $Q_{j}$ quantile estimator [41]:
  1. Choose an increasing sequence of proportions $\left\{q_{0},q_{1},\cdots ,q_{m-1},q_{m}\right\}$, where $q_{0}=0$ and $q_{m}=1$, with m≤n corresponding to the cumulative probabilities of each quantile.
  2. Estimate the quantiles as follows:
    (38) $$Q_{j}=X_{\left(I_{j}^{-}\right)}+\left(X_{\left(I_{j}^{+}\right)}-X_{\left(I_{j}^{-}\right)}\right)\left(I_{j}-I_{j}^{-}\right),\;j=1,\ldots ,\left(m-1\right).$$
    $I_{j}=q_{j}n+1/2$, $I_{j}^{-}$ is the largest integer less than or equal to $I_{j}$, and $I_{j}^{+}$ is the smallest integer greater than or equal to $I_{j}$.
- NO quantile estimator [42]:
  (39) $$\begin{array}{cc} {\mathrm{NO}}_{q}= & \;\left(B\left(0;n,q\right)2q+B\left(1;n,q\right)q\right)X_{\left(1\right)}+B\left(0;n,q\right)\left(2-3q\right)X_{\left(2\right)}-B\left(0;n,q\right)\left(1-q\right)X_{\left(3\right)}\\ & +\sum_{i=1}^{n-2}\left(B\left(i;n,q\right)\left(1-q\right)+B\left(i+1;n,q\right)q\right)X_{\left(i+1\right)}\\ & -B\left(n;n,q\right)qX_{\left(n-2\right)}+B\left(n;n,q\right)\left(3q-1\right)X_{\left(n-1\right)}\\ & +\left(B\left(n-1;n,q\right)\left(1-q\right)+B\left(n;n,q\right)\left(2-2q\right)\right)X_{\left(n\right)}. \end{array}$$
  B(i;n,q) is the binomial probability and *q* is the probability of success.

The first two methods are modifications of kernel-based quantile estimation methods, optimized by selecting the best bandwidth under various conditions. The last two methods adjust sample quantiles based on order statistics.

We apply these methods to estimate the MSE-RPs for P(I)(1,5), P(II)(0,1,5), P(III)(0,1,0.2), and P(IV)(0,1,0.1,0.5), and three sample sizes. Each method is tested through 1000 simulations, and the bias is reported in Table 1. Here, *n* represents the sample size, *m* represents the number of MSE-RPs, and the bias is calculated as follows:

$$ac=\mathrm{average}\left(\left|\hat{\mathrm{RPs}}-\mathrm{RPs}\right|\right).$$

The bold numbers in Table 1 indicate the minimum bias in each row. We analyze the impact of sample size, the number of MSE-RPs, the type of Pareto distribution, and parameter values on the accuracy of representative point estimation. From Table 1, it can be observed that, for fixed parameter values and a fixed number of MSE-RPs, the estimation bias decreases as the sample size increases. Conversely, as the number of MSE-RPs increases, the estimation bias increases for both quantile-based methods and the k-means method. However, the bias remains nearly unchanged for parameter-based estimation methods. This is because increasing the number of MSE-RPs does not introduce new estimators, ensuring that the estimation remains highly stable.

The type of Pareto distribution also significantly affects the bias of estimation methods in Table 1, mainly due to differences in distribution complexity and tail behavior. For Pareto I and II distributions, parameter-based estimation methods perform the best, as parameter estimation is relatively straightforward. However, for Pareto III and IV distributions, which introduce additional parameters, solving the nonlinear Equation (17) often results in convergence issues or requires large sample sizes, leading to increased bias. In contrast, the $B_{q}$ method utilizes quantile positions, which are more robust to heavy tails. The flexibility of Pareto III/IV in modeling the tail behavior aligns well with quantile-based estimation, thus reducing sensitivity to parameter misestimation.

The impact of parameters on MSE-RP estimation primarily manifests in the tail region. Smaller values of γ and α increase the difficulty of estimating μ, which relies heavily on the sample minimum. This, in turn, exacerbates the bias of parameter-based estimation methods. However, the $B_{q}$ method remains relatively stable, particularly for Pareto III and IV distributions, where the tail dominates. The $B_{q}$ method effectively captures the critical regions, improving the accuracy of MSE-RP estimation.

**Figure 1:**
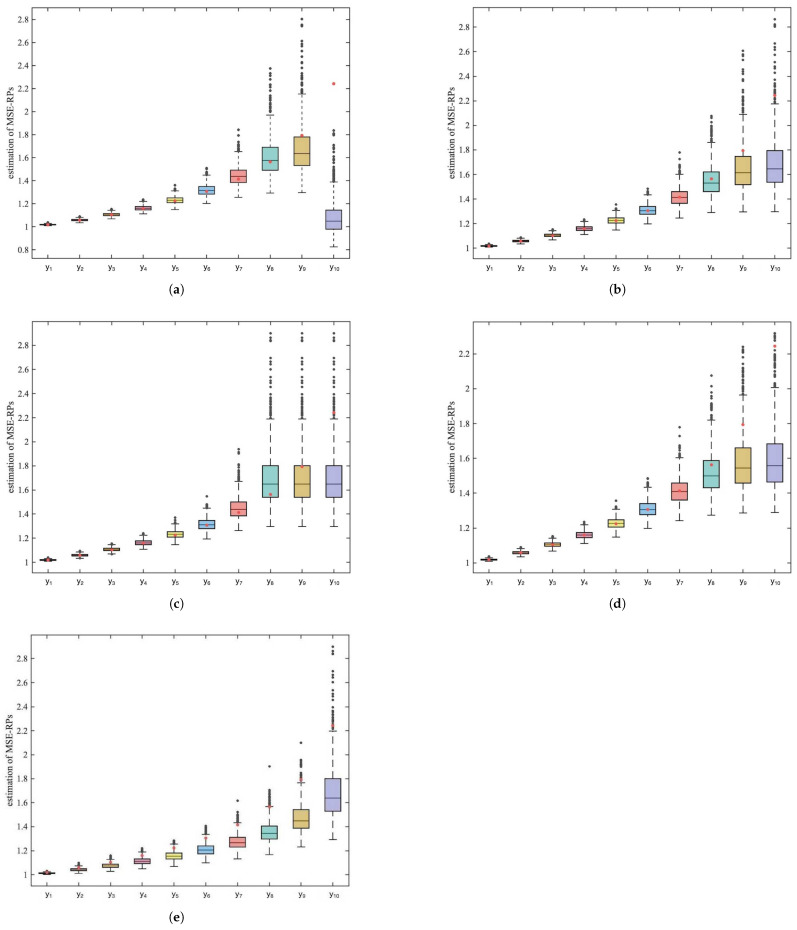
Box-plots of estimations of m=10 MSE-RPs from P(I)(1,5). (**a**) Description of the box-plots estimated for each representative point using the $HD_{q}$ quantile estimator. (**b**) Description of the box-plots estimated for each representative point using the $B_{q}$ quantile estimator. (**c**) Description of the box-plots estimated for each representative point using the $Q_{j}$ quantile estimator. (**d**) Description of the box-plots estimated for each representative point using the NO quantile estimator. (**e**) Description of the box-plots estimated for each representative point using the k-means method.

Furthermore, we analyze the sources of bias in quantile-based estimation. To investigate how different methods estimate MSE-RPs across various positions in the distribution, we plot the estimation results for m=10 MSE-RPs of P(I)(1,5) in Figure 1.

In Figure 1, the red dots represent the true values of the MSE-RPs. From the box-plots, it can be observed that for the k-means and $B_{q}$ methods, the estimation bias exhibits increasing fluctuation as the MSE-RPs move farther away from the mean of the Pareto distribution. Meanwhile, the $Q_{j}$ method shows low sensitivity to the estimation of MSE-RPs in the tail. The $HD_{q}$ method has a significant estimation bias for the last representative point, and the NO method exhibits a large overall bias with noticeable increases in fluctuation.

Therefore, considering all factors, the $B_{q}$ method is the most stable and accurate method for estimating the MSE-RPs of the Pareto distribution when γ and α are small. However, by examining the MSE-RPs and their corresponding probabilities, it can be observed that as the number of MSE-RPs increases, the probability associated with the tail MSE-RPs becomes significantly smaller, while their corresponding values become exceedingly large. Estimating these tail MSE-RPs notably increases the overall estimation bias.

Li et al. [30] previously proposed a method to measure the information gain (IG) of MSE-RPs, similar to the variance ratio used in principal component analysis. The range of IG is from 0 to 1, and the calculation formula is as follows:

(40) $$IG=1-\frac{MSE(y_{1},\ldots ,y_{k}|x)}{\mathrm{var}(x)},$$

where k≤m is the number of MSE-RPs used in calculating the IG. We can use the same IG function to calculate the number of MSE-RPs and their corresponding coverage under different levels of information gain. See Appendix C for details.

By calculating the information gain, we find that for the Pareto distribution, as the number of MSE-RPs increases, the information gain for the tail MSE-RPs becomes very small. For example, for the P(I)(1,5) distribution, when m=17, the first 12 MSE-RPs account for 90% of the information. We re-estimate the MSE-RPs for the above distributions with m=17 under different IG proportions, and the results are presented in Table 2.

The bold numbers in Table 2 indicate the minimum bias in each row. From the comparison between Table 1 and Table 2, it can be observed that sacrificing a small amount of information gain can significantly improve the accuracy of representative point estimation. Furthermore, when the parameters γ and α are small, the $B_{q}$ quantile method provides relatively accurate and stable estimates.

The IG truncation method dynamically determines the number of MSE-RPs required to achieve a specified IG level. This process focuses on selecting the most informative points while discarding tail points, which, although having high estimation bias, contribute little to the overall information. As a result, the outcomes presented in Table 2 can be considered robust to the choice of *m*.

In practical applications, the number of MSE-RPs needed can be determined by referring to Appendix C, which provides the number of MSE-RPs corresponding to different levels of IG and their coverage regions.

## 5. Real Data Study

### 5.1. Case I

We utilize commonly used Pareto I distribution data, which are taken from the appendix of Brazauskas’ study [43]. These data were also used by Kim et al. to demonstrate the best parameter estimator for the Pareto I distribution [44]. Therefore, we can directly use the estimated parameters of this dataset from their paper. The study indicates that the data follow a P(I)(497.085,1.2078) distribution, for which MSE-RPs do not exist.

Using the properties of the Pareto distribution discussed in Section 2 and Formula (6), this dataset can be approximately transformed into data following a P(I)(1,5) distribution, enabling the estimation of MSE-RPs. Since Formula (6) is an affine transformation that preserves the information content, no significant loss of information occurs during this process. To verify whether the data follow the distribution we mentioned, we performed a KS-test on both the transformed and original data, and the results were consistent. The p-value was 0.8943, and the KS statistic was 0.0472. Therefore, we believe the data follow the distribution mentioned above, and there is no loss of information before or after the transformation.

The transformed data are observed to have a distribution range of (1.0014,3.0836). Referring to the IG coverage tables provided in Appendix C, we perform the following estimations:

- For 90%≤IG≤95%, we first estimate m=7 MSE-RPs and select the first 6 MSE-RPs as those providing valid information.
- For 95%≤IG≤98%, we first estimate m=18 MSE-RPs and select the first 14 MSE-RPs as those providing valid information.

We compare the fit of the data using MSE-RPs estimated using different methods.

**Figure 2:**
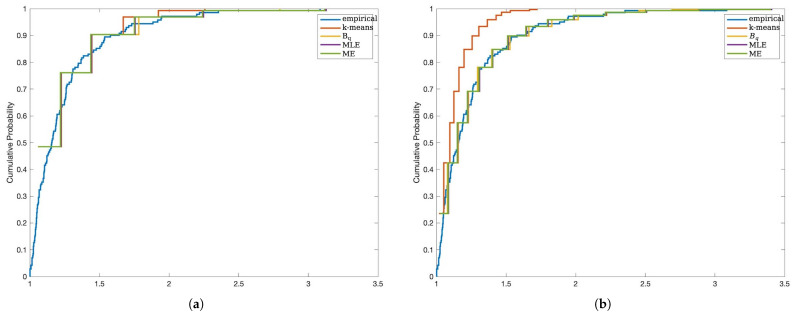
Case I data fitting P(I)(1,5) using MSE-RPs estimated using different methods. (**a**) Description of using MSE-RPs containing 90%≤IG≤95%. (**b**) Description of using MSE-RPs containing 95%≤IG≤98%.

From Figure 2, it can be observed that as IG increases, the empirical distribution fitted using MSE-RPs becomes closer to the true distribution. Furthermore, apart from the MSE-RPs estimated using the k-means method, which exhibit significant bias, the MSE-RPs estimated using the other three methods show no noticeable differences in the fitting process.

### 5.2. Case II

Next, we estimate MSE-RPs for real data from a Pareto IV distribution. The data were obtained from testing the unit stress voltage of miniature light bulbs [45]. According to the literature, the data follow a P(IV)(0,95.6575,0.3631,6.0973) distribution. Using the properties of the Pareto distribution and Formula (9), the data are transformed to correspond to a P(IV)(0,1,0.1,0.5) distribution. Since Formula (9) is also an affine transformation that preserves the information content, no significant loss of information occurs during this process. In order to confirm that the data follow the distribution we specified, we conducted a KS-test on both the original and transformed data. The results were consistent, with a *p*-value of 0.2004 and a KS statistic of 0.0957. So we conclude that the data follow the mentioned distribution, and the transformation process does not result in any loss of information.

The transformed data are observed to have a distribution range of (0.1187,1.9827). Referring to the IG coverage tables provided in Appendix C, we perform the following estimations:

- For 90%≤IG≤95%, we first estimate m=11 MSE-RPs and select the first 9 MSE-RPs as those providing valid information.
- For 95%≤IG≤98%, we first estimate m=20 MSE-RPs and select the first 16 MSE-RPs as those providing valid information.

We compare the fit of the data distribution using MSE-RPs estimated using different methods.

**Figure 3:**
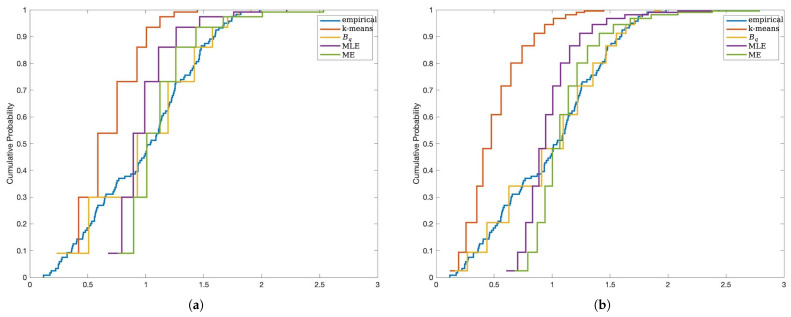
Case II data fitting P(IV)(0,1,0.1,0.5) using MSE-RPs estimated using different methods. (**a**) Description of using MSE-RPs containing 90%≤IG≤95%. (**b**) Description of using MSE-RPs containing 95%≤IG≤98%.

From Figure 3, it can be observed that, similarly, as IG increases, the empirical distribution fitted using MSE-RPs becomes closer to the true distribution. However, unlike Case I, in Case II, the MSE-RPs estimated using the $B_{q}$ method result in a closer fit to the true distribution in terms of empirical distribution, while the other three methods exhibit larger biases.

## 6. Conclusions

This paper primarily investigates the MSE-RPs of Pareto distributions and their estimation. We provided the uniqueness conditions for MSE-RPs and derived the corresponding unique intervals. For the computation of MSE-RPs of Pareto I and II distributions, we proposed an improved parametric k-means method.

For the estimation of MSE-RPs, we compared three categories of methods: the k-means method, the quantile-based estimation method, and methods that first estimate the parameters and then estimate the MSE-RPs. Based on simulations, we found that the k-means method is unsuitable for estimating MSE-RPs of Pareto distributions. The MLE-based parameter estimation method has the smallest bias when estimating MSE-RPs of Pareto I distributions, while the ME-based parameter estimation method performs best for Pareto II distributions. As the parameters γ and α decrease, the bias when using the $B_{q}$ quantile method to estimate MSE-RPs becomes minimal.

Since the tail MSE-RPs of Pareto distributions contribute significant bias while having very low probabilities, we propose using the IG function to calculate the information content of MSE-RPs. We also provide the number of MSE-RPs required for three levels of IG and their corresponding covered ranges to help select an appropriate number of MSE-RPs in practical analyses. Simulation results demonstrate that sacrificing a small amount of information gain can significantly improve the accuracy of representative point estimation.

Additionally, we applied the proposed methods to estimate MSE-RPs for real data from two different distributions. Using different IG intervals, we estimated MSE-RPs for the data and used them to fit the empirical distribution functions of the real data. These results were consistent with the simulation findings.

In conclusion, this paper studies the MSE-RPs of Pareto distributions and their estimation methods, analyzes the bias characteristics of representative point estimation, and proposes an IG-based optimization method for selecting MSE-RPs. The effectiveness and applicability of the proposed method were validated through simulations and real data studies.

## Figures and Tables

**Table 1:** Comparison of mean absolute bias from 1000 simulations in MSE-RP estimation based on sample sizes (n=20,50,100) and the number of MSE-RPs (m=5,10,17) for four types of Pareto distributions using different methods.

P(I)(1,5)								
		k-means	$HD_{q}$	$B_{q}$	$Q_{j}$	NO	MLE	ME
n = 20	m = 5	0.6244	0.7308	0.5248	0.5949	0.5831	**0.0215**	0.0926
	m = 10	1.1965	1.3143	0.9585	0.9729	1.0274	**0.0260**	0.1136
n = 50	m = 5	0.4999	0.4832	0.4027	0.4299	0.4524	**0.0084**	0.0588
	m = 10	1.0495	1.1282	0.8115	0.8270	0.8827	**0.0098**	0.0734
	m = 17	1.5442	1.4437	1.1951	1.2309	1.2693	**0.0122**	0.0830
n = 100	m = 5	0.4330	0.3619	0.3340	0.4179	0.3675	**0.0040**	0.0403
	m = 10	0.9402	0.9950	0.7154	0.7582	0.7707	**0.0005**	0.0165
	m = 17	1.4375	1.3352	1.0844	1.1220	1.1584	**0.0061**	0.0582
P(II)(0,1,5)								
		k-means	$HD_{q}$	$B_{q}$	$Q_{j}$	NO	MLE	ME
n = 20	m = 5	0.6327	0.6391	0.5318	0.5909	0.5881	0.3004	**0.2449**
	m = 10	1.2019	1.1455	0.9519	0.9672	1.0334	0.4343	**0.3501**
n = 50	m = 5	0.5001	0.4517	0.4005	0.4242	0.4579	0.1676	**0.1480**
	m = 10	1.0482	1.0016	0.8047	0.8201	0.8769	0.2341	**0.2215**
	m = 17	1.2133	1.2449	0.9206	0.9530	0.9812	0.3084	**0.2775**
n = 100	m = 5	0.4191	0.3445	0.3212	0.4027	0.3611	0.1116	**0.1074**
	m = 10	0.9367	0.8971	0.7084	0.7551	0.7698	0.1646	**0.1542**
	m = 17	1.1286	1.1215	0.8195	0.8511	0.8836	0.2091	**0.1985**
P(III)(0,1,0.2)								
		k-means	$HD_{q}$	$B_{q}$	$Q_{j}$	NO	MLE	ME
n = 20	m = 5	0.4507	0.3973	**0.3499**	0.3798	0.3850	0.4682	0.5153
	m = 10	0.8443	0.8867	0.6548	0.6767	0.7055	**0.4795**	0.5273
n = 50	m = 5	0.3491	0.2579	**0.2463**	0.2586	0.2781	0.4039	0.4199
	m = 10	0.7155	0.7263	0.5196	0.5378	0.5706	**0.4092**	0.4314
	m = 17	1.0939	1.0846	0.8075	0.8303	0.8636	**0.4078**	0.4300
n = 100	m = 5	0.3079	0.1938	**0.1867**	0.1975	0.2059	0.4682	0.5153
	m = 10	0.6184	0.6178	0.4380	0.4727	0.4813	**0.3542**	0.3651
	m = 17	0.9937	0.9762	0.7107	0.7345	0.7623	**0.3617**	0.3726
P(IV)(0,1,0.1,0.5)								
		k-means	$HD_{q}$	$B_{q}$	$Q_{j}$	NO	MLE	ME
n = 20	m = 5	0.5168	0.5184	**0.4140**	0.4325	0.4533	0.7317	0.7478
	m = 10	0.9466	1.0216	**0.7395**	0.7775	0.8026	0.7453	0.7638
n = 50	m = 5	0.4065	0.3319	**0.3038**	0.3693	0.3374	0.6847	0.6920
	m = 10	0.8222	0.8568	**0.6170**	0.6563	0.6682	0.6844	0.6917
	m = 17	1.0939	1.0846	0.8075	0.8303	0.8636	**0.6726**	0.6849
n = 100	m = 5	0.3566	0.2479	**0.2382**	0.2404	0.2641	0.6376	0.6418
	m = 10	0.7219	0.7425	**0.5237**	0.5565	0.5741	0.6381	0.6431
	m = 17	0.9937	0.9762	0.7107	0.7345	0.7623	**0.6412**	0.6470

**Table 2:** Comparison of mean absolute bias from 1000 simulations in MSE-RP estimation at three levels of IG with m=17.

P(I)(1,5)								
		k-means	$HD_{q}$	$B_{q}$	$Q_{j}$	NO	MLE	ME
90%≤IG≤95%	n = 100	0.3677	0.1373	0.1145	0.1796	0.1147	**0.0033**	0.0321
	n = 50	0.4245	0.1725	0.1534	0.2062	0.1583	**0.0067**	0.0458
95%≤IG≤98%	n = 100	0.5693	0.2572	0.2333	0.2898	0.2552	**0.0039**	0.0375
	n = 50	0.6434	0.3426	0.3029	0.3466	0.3294	**0.0079**	0.0535
98%≤IG	n = 100	0.9768	0.8248	0.5915	0.6400	0.6532	**0.0049**	0.0475
	n = 50	1.0733	0.9683	0.6977	0.7355	0.7558	**0.0100**	0.0678
P(II)(0,1,5)								
		k-means	$HD_{q}$	$B_{q}$	$Q_{j}$	NO	MLE	ME
90%≤IG≤95%	n = 100	0.3684	0.1347	0.1172	0.1723	0.1195	0.0671	**0.0643**
	n = 50	0.4244	0.1773	0.1577	0.2112	0.1644	0.0988	**0.0901**
95%≤IG≤98%	n = 100	0.5708	0.2581	0.2412	0.2879	0.2645	0.0966	**0.0922**
	n = 50	0.6430	0.3354	0.3053	0.3492	0.3367	0.1424	**0.1290**
98%≤IG	n = 100	0.9805	0.7551	0.6055	0.6455	0.6630	0.1512	**0.1438**
	n = 50	1.0725	0.8693	0.7017	0.7396	0.7624	0.2229	**0.2011**
P(III)(0,1,0.2)								
		k-means	$HD_{q}$	$B_{q}$	$Q_{j}$	NO	MLE	ME
90%≤IG≤95%	n = 100	0.3074	0.1055	**0.0920**	0.1225	0.0921	0.3624	0.3682
	n = 50	0.3525	0.1324	**0.1213**	0.1539	0.1268	0.4078	0.4206
95%≤IG≤98%	n = 100	0.3817	0.1469	**0.1331**	0.1638	0.1382	0.3618	0.3682
	n = 50	0.4363	0.1820	**0.1723**	0.2013	0.1866	0.4065	0.4207
98%≤IG	n = 100	0.6628	0.4709	0.3625	0.3878	0.3964	**0.3604**	0.3689
	n = 50	0.7429	0.5813	0.4387	0.4629	0.4809	**0.4045**	0.4227
P(IV)(0,1,0.1,0.5)								
		k-means	$HD_{q}$	$B_{q}$	$Q_{j}$	NO	MLE	ME
90%≤IG≤95%	n = 100	0.3424	0.1203	**0.1061**	0.1497	0.1094	0.6424	0.6457
	n = 50	0.3816	0.1615	**0.1454**	0.1898	0.1527	0.6781	0.6850
95%≤IG≤98%	n = 100	0.4310	0.1691	**0.1563**	0.1964	0.1694	0.6422	0.6459
	n = 50	0.4785	0.2248	**0.2082**	0.2486	0.2257	0.6773	0.6848
98%≤IG	n = 100	0.7559	0.5726	**0.4279**	0.4615	0.4746	0.6417	0.6464
	n = 50	0.8246	0.7006	**0.5137**	0.5482	0.5595	0.6748	0.6844

## Data Availability

The data used in Case I were obtained from Table A2 in the Appendix of Reference [43]. The data used in Case II were obtained from Table 1 in Section 6 of reference [13]. Further details about the data sources can be found in the cited references. No new data were created in this study.

## Appendix A. Proof

### Appendix A.1

**Proof** **of** **Lemma** **1.** Taking the logarithm of the density function of P(IV)(0,1,γ,α), we have

(A1) $$g\left(x\right)=\log f\left(x\right)=\log\alpha +\log\gamma -\left(\alpha +1\right)\log\left(1+x^{\frac{1}{\gamma }}\right)+\left(\frac{1}{\gamma }-1\right)\log x.$$

By taking the second derivative of g(x), we obtain

(A2) $$\frac{\partial^{2}g\left(x\right)}{\partial x^{2}}=\frac{1}{x^{2}}\left[\frac{\left(\alpha +1\right)}{\gamma^{2}}{\left(\frac{x^{1/\gamma }}{1+x^{1/\gamma }}\right)}^{2}-\frac{\left(\alpha +1\right)}{\gamma }\left(\frac{1}{\gamma }-1\right)\left(\frac{x^{1/\gamma }}{1+x^{1/\gamma }}\right)-\left(\frac{1}{\gamma }-1\right)\right].$$

From (A2), it is evident that when $\frac{1}{\gamma }-1\leq 0$, we have $\frac{\partial^{2}g\left(x\right)}{\partial x^{2}}\geq 0$, which implies the function is not log-concave. Therefore, it is required that $\frac{1}{\gamma }>1$. Let $z=\frac{x^{1/\gamma }}{1+x^{1/\gamma }},\;0<z<1$, then

(A3) $$h\left(z\right)=\frac{\left(\alpha +1\right)}{\gamma^{2}}z^{2}-\frac{\left(\alpha +1\right)}{\gamma }\left(\frac{1}{\gamma }-1\right)z-\left(\frac{1}{\gamma }-1\right).$$

It is clear that h(z) is an upward-opening parabola, and *z* is an increasing function of *x*. The two roots of h(z)=0 are as follows:

(A4) $$\begin{array}{cc} z_{1}^{*} & =\frac{\left(\alpha +1\right)\left(\frac{1}{\gamma }-1\right)-\sqrt{{\left(\alpha +1\right)}^{2}{\left(\frac{1}{\gamma }-1\right)}^{2}+4\left(\alpha +1\right)\left(\frac{1}{\gamma }-1\right)}}{2(\alpha +1)/\gamma },\\ z_{2}^{*} & =\frac{\left(\alpha +1\right)\left(\frac{1}{\gamma }-1\right)+\sqrt{{\left(\alpha +1\right)}^{2}{\left(\frac{1}{\gamma }-1\right)}^{2}+4\left(\alpha +1\right)\left(\frac{1}{\gamma }-1\right)}}{2(\alpha +1)/\gamma }. \end{array}$$

Clearly, $z_{1}^{*}<0$ and $z_{2}^{*}>0$. Next, we examine whether $z_{2}^{*}$ is within the range of *z*:

(A5) $$z_{2}^{*}-1=-\frac{1}{2}\left(1+\gamma \right)+\frac{1}{2}\sqrt{{\left(1-\gamma \right)}^{2}+\frac{4(\gamma -\gamma^{2})}{\left(\alpha +1\right)}}.$$

To determine whether $z_{2}^{*}>1$ or $z_{2}^{*}<1$, we square both parts of (A5) and subtract the squares. Since

(A6) $${\left(1-\gamma \right)}^{2}+\frac{4(\gamma -\gamma^{2})}{\left(\alpha +1\right)}-{\left(1+\gamma \right)}^{2}=\frac{-4\gamma \alpha -4\gamma^{2}}{\alpha +1}<0,$$

it follows that $z_{2}^{*}<1$. Therefore, when $\frac{1}{\gamma }>1$, and *z* lies within $\left(0,z_{2}^{*}\right)$, the density function of P(IV)(0,1,γ,α) is log-concave, which guarantees the uniqueness of the MSE-RPs. Furthermore, as α→+∞, $z_{2}^{*}\to 1-\gamma$. □

### Appendix A.2

**Proof** **of** **Theorem** **1.** Let

(A7) $$G\left(y_{i+1}\right)=M_{i+1}F\left(M_{i+1}\right)-M_{i}F\left(M_{i}\right)-\int_{M_{i}}^{M_{i+1}}F\left(x\right)dx-y_{i}\left(F\left(M_{i+1}\right)-F\left(M_{i}\right)\right),$$

where $M_{1}=F^{-1}\left(0\right)$, $M_{i}=\frac{y_{i-1}+y_{i}}{2},\;i=2,\cdots ,m$, and $M_{m+1}=F^{-1}\left(1\right)$. To prove that the equation has a solution, for i=1,⋯,m−1, we need to show that $G(y_{i+1}=y_{i})<0$ and $\lim_{y_{i+1}\to +\infty }G\left(y_{i+1}\right)>0$. For i=m, we need to show that $G(y_{i+1}=y_{i})>0$ and $\lim_{y_{i+1}\to +\infty }G\left(y_{i+1}\right)<0$.

1. Case I: For i=1,⋯,m−1,
  (A8) $$G\left(y_{i+1}=y_{i}\right)=\left(y_{i}-M_{i}\right)F\left(M_{i}\right)-\int_{M_{i}}^{y_{i}}F\left(x\right)dx.$$
  By the mean value theorem, there exists $\eta \in (M_{i},y_{i})$ such that $\int_{M_{i}}^{y_{i}}F\left(x\right)dx=\left(y_{i}-M_{i}\right)F\left(\eta \right)$. Therefore, $G(y_{i+1}=y_{i})<0$.
  (A9) $$\lim_{y_{i+1}\to +\infty }G\left(y_{i+1}\right)=\lim_{M_{i+1}\to +\infty }\left(y_{i}-M_{i}\right)F\left(M_{i}\right)+\left(M_{i+1}-y_{i}\right)F\left(M_{i+1}\right)-\int_{M_{i}}^{M_{i+1}}F\left(x\right)dx.$$
  Substituting $F\left(x\right)=1-{\left(1+x^{1/\gamma }\right)}^{-\alpha }$, we have
  (A10) $$\begin{array}{cc} h\left(y_{i}\right)=\lim_{y_{i+1}\to +\infty }G\left(y_{i+1}\right) & =\left(y_{i}-M_{i}\right)\left(F\left(M_{i}\right)-1\right)+\int_{M_{i}}^{+\infty }\left(1-F\left(x\right)\right)dx\\ & =y_{i}\left(F\left(M_{i}\right)-1\right)+\int_{M_{i}}^{+\infty }xf\left(x\right)dx, \end{array}$$
  where the last equality holds because, when $\frac{\alpha }{\gamma }>2$,
  (A11) $$\lim_{x\to +\infty }x\left(F\left(x\right)-1\right)=\lim_{x\to +\infty }x^{1-\frac{\alpha }{\gamma }}=0.$$
  For i=1,
  (A12) $$h\left(y_{1}\right)=-y_{1}+\int_{0}^{+\infty }xf\left(x\right)dx=E\left(X\right)-y_{1}.$$
  Thus, when $y_{1}<E\left(X\right)$, $h(y_{1})>0$, which ensures the existence of a solution. For i=2,⋯,m−1,
  (A13) $$\begin{array}{cc} h(y_{i}) & =y_{i}\left(F\left(M_{i}\right)-1\right)+\int_{0}^{+\infty }xf\left(x\right)dx-\sum_{j=1}^{i-1}\int_{M_{j}}^{M_{j+1}}xf\left(x\right)dx\\ & =y_{i}\left(F\left(M_{i}\right)-1\right)+E\left(X\right)-\sum_{j=1}^{i-1}y_{j}. \end{array}$$
  Thus, when $y_{i}<\frac{E\left(X\right)-\sum_{j=1}^{i-1}y_{j}}{1-F(M_{i})}$, $h(y_{i})>0$, and the equation has a solution.
2. Case II: For i=m
  (A14) $$G\left(y_{m}=y_{m-1}\right)=\lim_{N\to +\infty }\left(N-y_{m-1}\right)F\left(N\right)-\int_{y_{m-1}}^{N}F\left(x\right)dx.$$
  By the mean value theorem, there exists $\eta \in (y_{m-1},N)$ such that $\int_{y_{m-1}}^{N}F\left(x\right)dx=\left(N-y_{m-1}\right)F\left(\eta \right)$. Thus, $G(y_{m}=y_{m-1})>0$.
  (A15) $$\lim_{y_{m}\to +\infty }G\left(y_{m}\right)=\lim_{M_{m+1}\to +\infty }-y_{m}\left(F\left(M_{m+1}\right)-F\left(M_{m}\right)\right)<0.$$

□

## Appendix B. MSE-RPs of Pareto III and IV Distributions

**Table A1:** The points of MSE-RPs from P(III)(0,1,0.2).

	MSE	RP1	RP2	RP3	RP4	RP5	RP6	RP7	RP8	RP9	RP10	RP11	RP12	RP13	RP14	RP15	RP16
m = 2	0.081005705	0.893248503	1.624569032														
m = 3	0.046924	0.792100	1.264540	2.150314													
m = 4	0.030749	0.722458	1.095883	1.588422	2.665352												
m = 5	0.021748	0.670249	0.990624	1.343028	1.899748	3.175156											
m = 6	0.016210	0.629031	0.915762	1.198240	1.573247	2.206232	3.681976										
m = 7	0.012554	0.595321	0.858417	1.099461	1.385825	1.796710	2.510388	4.186923									
m = 8	0.010013	0.567032	0.812355	1.026063	1.261175	1.564830	2.016989	2.813247	4.690613								
m = 9	0.008174	0.542819	0.774120	0.968406	1.170558	1.412900	1.739604	2.235580	3.115306	5.193419							
m = 10	0.006800	0.521766	0.741610	0.921328	1.100659	1.304042	1.559465	1.912082	2.453194	3.416831	5.695577						
m = 11	0.005746	0.503227	0.713457	0.881789	1.044432	1.221182	1.431611	1.703127	2.083222	2.670200	3.717980	6.197247					
m = 12	0.004920	0.486728	0.688722	0.847862	0.997781	1.155313	1.335200	1.555742	1.845052	2.253536	2.886806	4.018854	6.698539				
m = 13	0.004260	0.471912	0.666734	0.818261	0.958149	1.101222	1.259236	1.445329	1.677757	1.985886	2.423314	3.103136	4.319518	7.199533			
m = 14	0.003725	0.458507	0.646998	0.792087	0.923850	1.055678	1.197364	1.358900	1.553005	1.798412	2.126004	2.592731	3.319267	4.620018	7.700288		
m = 15	0.003285	0.446297	0.629138	0.768688	0.893721	1.016564	1.145654	1.288950	1.455822	1.659071	1.918161	2.265638	2.761895	3.535249	4.920388	8.200847	
m = 16	0.002918	0.435111	0.612864	0.747579	0.866934	0.982432	1.101539	1.230836	1.377547	1.550909	1.764044	2.037287	2.404934	2.930878	3.751119	5.220652	8.701244
m = 17	0.002610	0.424810	0.597944	0.728389	0.842877	0.952257	1.063273	1.181533	1.312824	1.464112	1.644728	1.868254	2.155975	2.543988	3.099726	3.966900	5.520828
m = 18	0.002348	0.415283	0.584195	0.710828	0.821087	0.925287	1.029620	1.138984	1.258163	1.392609	1.549251	1.737649	1.971919	2.274345	2.682864	3.268472	4.182610
m = 19	0.002124	0.406433	0.571465	0.694667	0.801208	0.900961	0.999682	1.101739	1.211192	1.332442	1.470827	1.633366	1.829920	2.075187	2.392483	2.821608	3.437139
m = 20	0.001930	0.398184	0.559631	0.679718	0.782960	0.878848	0.972791	1.068747	1.170240	1.280923	1.405031	1.547906	1.716732	1.921712	2.178159	2.510446	2.960250
m = 21	0.001762	0.390469	0.548588	0.665831	0.766116	0.858610	0.948434	1.039225	1.134101	1.236160	1.348856	1.476378	1.624142	1.799537	2.013145	2.280909	2.628276
m = 22	0.001614	0.383232	0.538250	0.652880	0.750496	0.839981	0.926216	1.012580	1.101874	1.196783	1.300186	1.415454	1.546794	1.699742	1.881919	2.104304	2.383489
m = 23	0.001485	0.376424	0.528543	0.640759	0.735950	0.822744	0.905821	0.988349	1.072880	1.161777	1.257491	1.362796	1.481044	1.616503	1.774855	1.963975	2.195251
m = 24	0.001370	0.370003	0.519402	0.629380	0.722352	0.806724	0.886999	0.966171	1.046591	1.130371	1.219635	1.316709	1.424326	1.545859	1.685666	1.849592	2.045778
m = 25	0.001269	0.363935	0.510773	0.618665	0.709599	0.791774	0.869544	0.945754	1.022593	1.101971	1.185757	1.275939	1.374784	1.485021	1.610073	1.754403	1.924033
m = 26	0.001178	0.358186	0.502610	0.608552	0.697601	0.777774	0.853287	0.926865	1.000556	1.076111	1.155196	1.239534	1.331043	1.431969	1.545062	1.673812	1.822802
m = 27	0.001097	0.352729	0.494869	0.598982	0.686283	0.764620	0.838090	0.909309	0.980212	1.052419	1.127429	1.206762	1.292060	1.385207	1.488451	1.604584	1.737175
m = 28	0.001023	0.347541	0.487516	0.589908	0.675580	0.752225	0.823833	0.892927	0.961344	1.030594	1.102044	1.177046	1.257031	1.343599	1.438623	1.544373	1.663692
m = 29	0.000957	0.342599	0.480518	0.581285	0.665436	0.740515	0.810419	0.877586	0.943772	1.010392	1.078707	1.149931	1.225327	1.306273	1.394351	1.491441	1.599845
m = 30	0.000897	0.337883	0.473846	0.573078	0.655800	0.729425	0.797761	0.863172	0.927344	0.991612	1.057145	1.125049	1.196447	1.272543	1.354691	1.444469	1.543774
m = 31	0.000843	0.333378	0.467476	0.565251	0.646630	0.718899	0.785786	0.849591	0.911934	0.974085	1.037136	1.102100	1.169989	1.241865	1.318901	1.402441	1.494069
		RP17	RP18	RP19	RP20	RP21	RP22	RP23	RP24	RP25	RP26	RP27	RP28	RP29	RP30	RP31	
m = 17		9.201507															
m = 18		5.820932	9.701655														
m = 19		4.398262	6.120974	10.201706													
m = 20		3.605744	4.613867	6.420963	10.701674												
m = 21		3.098813	3.774299	4.829431	6.720907	11.201569											
m = 22		2.746003	3.237314	3.942814	5.044962	7.020812	11.701401										
m = 23		2.485936	2.863649	3.375766	4.111295	5.260464	7.320682	12.201178									
m = 24		2.286034	2.588279	2.981231	3.514177	4.279747	5.475941	7.620523	12.700906								
m = 25		2.127382	2.376686	2.690539	3.098762	3.652556	4.448176	5.691397	7.920337	13.200591							
m = 26		1.998240	2.208827	2.467235	2.792733	3.216251	3.790908	4.616585	5.906833	8.220127	13.700237						
m = 27		1.890933	2.072259	2.290146	2.557699	2.894874	3.333705	3.929236	4.784977	6.122252	8.519896	14.199850					
m = 28		1.800235	1.958847	2.146128	2.371361	2.648096	2.996970	3.451131	4.067546	4.953354	6.337657	8.819647	14.699431				
m = 29		1.722464	1.863050	2.026586	2.219873	2.452493	2.738437	3.099030	3.568534	4.205839	5.121717	6.553048	9.119380	15.198984			
m = 30		1.654949	1.780963	1.925665	2.094181	2.293518	2.533556	2.828733	3.201060	3.685917	4.344118	5.290069	6.768427	9.419099	15.698512		
m = 31		1.595710	1.709754	1.839238	1.988116	2.161658	2.367080	2.614562	2.918990	3.303065	3.803283	4.482385	5.458410	6.983796	9.718803	16.198018	

**Table A2:** The corresponding probabilities of MSE-RPs from P(III)(0,1,0.2).

	P1	P2	P3	P4	P5	P6	P7	P8	P9	P10	P11	P12	P13	P14	P15	P16
m = 2	0.759735	0.240265														
m = 3	0.534851	0.400680	0.064469													
m = 4	0.383170	0.430096	0.164274	0.022460												
m = 5	0.283123	0.400708	0.234239	0.072513	0.009417											
m = 6	0.215635	0.353219	0.267478	0.123827	0.035341	0.004501										
m = 7	0.168676	0.304742	0.274248	0.163070	0.068132	0.018763	0.002369									
m = 8	0.134990	0.261237	0.265497	0.187507	0.099284	0.039460	0.010681	0.001343								
m = 9	0.110152	0.224090	0.248945	0.199247	0.124372	0.061963	0.023989	0.006435	0.000807							
m = 10	0.091389	0.192998	0.229277	0.201662	0.142152	0.082844	0.039889	0.015218	0.004061	0.000509						
m = 11	0.076910	0.167141	0.209103	0.197859	0.153120	0.100258	0.056114	0.026481	0.010016	0.002665	0.000334					
m = 12	0.065529	0.145634	0.189765	0.190239	0.158475	0.113563	0.071070	0.038801	0.018086	0.006804	0.001807	0.000226				
m = 13	0.056437	0.127683	0.171881	0.180501	0.159545	0.122862	0.083860	0.050991	0.027400	0.012671	0.004751	0.001260	0.000158			
m = 14	0.049069	0.112623	0.155667	0.169784	0.157524	0.128628	0.094123	0.062225	0.037124	0.019742	0.009082	0.003398	0.000901	0.000113		
m = 15	0.043020	0.099912	0.141131	0.158819	0.153385	0.131472	0.101855	0.072015	0.046592	0.027443	0.014491	0.006643	0.002482	0.000658	0.000082	
m = 16	0.038000	0.089119	0.128177	0.148056	0.147879	0.132010	0.107256	0.080146	0.055336	0.035269	0.020590	0.010819	0.004948	0.001846	0.000489	0.000061
m = 17	0.033789	0.079895	0.116667	0.137757	0.141558	0.130792	0.110628	0.086584	0.063072	0.042822	0.027006	0.015667	0.008203	0.003745	0.001397	0.000370
m = 18	0.030226	0.071967	0.106447	0.128061	0.134823	0.128287	0.112300	0.091421	0.069659	0.049821	0.033418	0.020915	0.012078	0.006309	0.002877	0.001072
m = 19	0.027186	0.065111	0.097369	0.119031	0.127952	0.124871	0.112592	0.094810	0.075065	0.056086	0.039578	0.026313	0.016376	0.009426	0.004914	0.002239
m = 20	0.024573	0.059150	0.089294	0.110677	0.121135	0.120843	0.111794	0.096937	0.079329	0.061526	0.045305	0.031651	0.020904	0.012956	0.007438	0.003873
m = 21	0.022311	0.053941	0.082098	0.102986	0.114497	0.116431	0.110156	0.097993	0.082534	0.066110	0.050483	0.036766	0.025493	0.016753	0.010350	0.005931
m = 22	0.020340	0.049365	0.075671	0.095923	0.108119	0.111808	0.107889	0.098159	0.084788	0.069855	0.055047	0.041539	0.030000	0.020681	0.013539	0.008344
m = 23	0.018614	0.045327	0.069918	0.089448	0.102046	0.107104	0.105165	0.097603	0.086209	0.072807	0.058973	0.045890	0.034313	0.024620	0.016897	0.011029
m = 24	0.017095	0.041749	0.064753	0.083516	0.096301	0.102411	0.102124	0.096470	0.086912	0.075030	0.062267	0.049773	0.038352	0.028472	0.020325	0.013901
m = 25	0.015750	0.038564	0.060106	0.078082	0.090894	0.097797	0.098876	0.094886	0.087009	0.076599	0.064954	0.053168	0.042059	0.032157	0.023738	0.016878
m = 26	0.014554	0.035719	0.055913	0.073103	0.085820	0.093308	0.095508	0.092958	0.086603	0.077590	0.067074	0.056075	0.045401	0.035618	0.027065	0.019889
m = 27	0.013487	0.033168	0.052121	0.068536	0.081071	0.088974	0.092087	0.090774	0.085784	0.078079	0.068674	0.058509	0.048362	0.038815	0.030249	0.022871
m = 28	0.012530	0.030872	0.048682	0.064345	0.076633	0.084816	0.088665	0.088409	0.084632	0.078136	0.069806	0.060495	0.050940	0.041722	0.033249	0.025772
m = 29	0.011670	0.028800	0.045557	0.060493	0.072490	0.080843	0.085279	0.085920	0.083216	0.077828	0.070522	0.062064	0.053143	0.044325	0.036033	0.028550
m = 30	0.010893	0.026924	0.042709	0.056949	0.068624	0.077062	0.081958	0.083358	0.081595	0.077215	0.070873	0.063251	0.054986	0.046622	0.038584	0.031175
m = 31	0.010190	0.025220	0.040108	0.053685	0.065017	0.073471	0.078724	0.080759	0.079820	0.076349	0.070906	0.064092	0.056492	0.048617	0.040889	0.033622
	P17	P18	P19	P20	P21	P22	P23	P24	P25	P26	P27	P28	P29	P30	P31	
m = 17	0.000046															
m = 18	0.000284	0.000036														
m = 19	0.000834	0.000221	0.000028													
m = 20	0.001763	0.000657	0.000174	0.000022												
m = 21	0.003085	0.001404	0.000523	0.000138	0.000017											
m = 22	0.004775	0.002482	0.001129	0.000420	0.000111	0.000014										
m = 23	0.006785	0.003878	0.002014	0.000916	0.000341	0.000090	0.000011									
m = 24	0.009052	0.005561	0.003176	0.001649	0.000749	0.000279	0.000074	0.000009								
m = 25	0.011511	0.007483	0.004591	0.002620	0.001360	0.000618	0.000230	0.000061	0.000008							
m = 26	0.014097	0.009593	0.006227	0.003817	0.002177	0.001130	0.000513	0.000191	0.000051	0.000006						
m = 27	0.016747	0.011839	0.008042	0.005214	0.003194	0.001821	0.000944	0.000429	0.000160	0.000042	0.000005					
m = 28	0.019408	0.014169	0.009996	0.006781	0.004392	0.002689	0.001532	0.000795	0.000361	0.000134	0.000036	0.000004				
m = 29	0.022033	0.016538	0.012045	0.008483	0.005748	0.003720	0.002276	0.001297	0.000672	0.000305	0.000114	0.000030	0.000004			
m = 30	0.024584	0.018904	0.014151	0.010287	0.007234	0.004897	0.003167	0.001937	0.001104	0.000572	0.000260	0.000097	0.000026	0.000003		
m = 31	0.027029	0.021231	0.016277	0.012158	0.008823	0.006198	0.004192	0.002710	0.001657	0.000944	0.000489	0.000222	0.000083	0.000022	0.000003	

**Table A3:** The points of MSE-RPs from P(IV)(0,1,0.1,0.5).

	MSE	RP1	RP2	RP3	RP4	RP5	RP6	RP7	RP8	RP9	RP10	RP11	RP12	RP13	RP14	RP15	RP16
m = 2	0.060997	1.073328	1.797978														
m = 3	0.035699	1.001741	1.421058	2.369184													
m = 4	0.023552	0.950106	1.259380	1.756498	2.927609												
m = 5	0.016738	0.909934	1.165271	1.499730	2.087874	3.479813											
m = 6	0.012520	0.877230	1.101445	1.355579	1.736728	2.417078	4.028470										
m = 7	0.009724	0.849773	1.053992	1.261844	1.541057	1.972432	2.744937	4.574898									
m = 8	0.007773	0.826198	1.016533	1.195035	1.415336	1.725089	2.207414	3.071896	5.119828								
m = 9	0.006357	0.805607	0.985714	1.144306	1.327099	1.566758	1.908503	2.441918	3.398221	5.663702							
m = 10	0.005296	0.787374	0.959595	1.103973	1.261260	1.456271	1.717401	2.091572	2.676082	3.724081	6.206803						
m = 11	0.004481	0.771051	0.936964	1.070775	1.209858	1.374446	1.584345	1.867679	2.274413	2.909991	4.049591	6.749318					
m = 12	0.003841	0.756303	0.917022	1.042710	1.168304	1.311128	1.486132	1.711938	2.017753	2.457091	3.143704	4.374829	7.291381				
m = 13	0.003329	0.742874	0.899212	1.018482	1.133771	1.260437	1.410459	1.597154	1.839288	2.167695	2.639643	3.377263	4.699852	7.833086			
m = 14	0.002913	0.730564	0.883135	0.997212	1.104427	1.218740	1.350190	1.508903	1.707853	1.966500	2.317542	2.822096	3.610697	5.024702	8.374504		
m = 15	0.002571	0.719216	0.868495	0.978282	1.079037	1.183673	1.300901	1.438808	1.606909	1.818377	2.093623	2.467316	3.004468	3.844030	5.349412	8.915686	
m = 16	0.002286	0.708702	0.855064	0.961247	1.056733	1.153635	1.259709	1.381673	1.526852	1.704682	1.928799	2.220684	2.617033	3.186775	4.077279	5.674005	9.456674
m = 17	0.002045	0.698916	0.842665	0.945772	1.036893	1.127505	1.224656	1.334104	1.461720	1.614588	1.802324	2.039155	2.347697	2.766704	3.369027	4.310458	5.998500
m = 18	0.001841	0.689774	0.831159	0.931604	1.019055	1.104474	1.194366	1.293790	1.407615	1.541370	1.702149	1.899885	2.149465	2.474674	2.916335	3.551232	4.543578
m = 19	0.001666	0.681201	0.820431	0.918545	1.002873	1.083947	1.167846	1.259107	1.361880	1.480630	1.620792	1.789607	1.997395	2.259742	2.601620	3.065934	3.733397
m = 20	0.001515	0.673137	0.810387	0.906441	0.988078	1.065474	1.144362	1.228880	1.322646	1.429371	1.553357	1.700079	1.877000	2.094869	2.369992	2.728541	3.215504
m = 21	0.001383	0.665531	0.800951	0.895164	0.974460	1.048709	1.123361	1.202238	1.288558	1.385480	1.496506	1.625910	1.779281	1.964349	2.192316	2.480221	2.855441
m = 22	0.001268	0.658336	0.792057	0.884612	0.961854	1.033383	1.104417	1.178524	1.258612	1.347425	1.447886	1.563423	1.698354	1.858430	2.051670	2.289743	2.590433
m = 23	0.001167	0.651516	0.783649	0.874702	0.950123	1.019283	1.087199	1.157233	1.232046	1.314068	1.405790	1.510027	1.630204	1.770730	1.937543	2.138968	2.387155
m = 24	0.001077	0.645035	0.775682	0.865362	0.939159	1.006237	1.071444	1.137969	1.208276	1.284548	1.368948	1.463838	1.571998	1.696896	1.843059	2.016630	2.226251
m = 25	0.000997	0.638865	0.768113	0.856532	0.928870	0.994106	1.056942	1.120420	1.186845	1.258201	1.336399	1.423458	1.521682	1.633859	1.763530	1.915357	2.095698
m = 26	0.000926	0.632979	0.760907	0.848163	0.919180	0.982776	1.043521	1.104335	1.167389	1.234508	1.307402	1.387827	1.477726	1.579390	1.695647	1.830124	1.987632
m = 27	0.000862	0.627356	0.754034	0.840210	0.910026	0.972151	1.031042	1.089511	1.149617	1.213056	1.281375	1.356125	1.438973	1.531833	1.637009	1.757384	1.896690
m = 28	0.000805	0.621974	0.747467	0.832636	0.901354	0.962153	1.019389	1.075782	1.133294	1.193513	1.257857	1.327710	1.404526	1.489929	1.585829	1.694564	1.819087
m = 29	0.000753	0.616815	0.741181	0.825408	0.893117	0.952714	1.008465	1.063009	1.118225	1.175611	1.236477	1.302073	1.373683	1.452707	1.540754	1.639750	1.752077
m = 30	0.000706	0.611864	0.735154	0.818497	0.885274	0.943778	0.998189	1.051078	1.104251	1.159129	1.216933	1.278803	1.345886	1.419405	1.500735	1.591488	1.693618
m = 31	0.000663	0.607106	0.729369	0.811879	0.877792	0.935294	0.988493	1.039892	1.091239	1.143885	1.198977	1.257566	1.320686	1.389417	1.464951	1.548656	1.642158
		RP17	RP18	RP19	RP20	RP21	RP22	RP23	RP24	RP25	RP26	RP27	RP28	RP29	RP30	RP31	
m = 17		9.997500															
m = 18		6.322913	10.538188														
m = 19		4.776647	6.647255	11.078759													
m = 20		3.915529	5.009672	6.971538	11.619229												
m = 21		3.365050	4.097631	5.242661	7.295767	12.159612											
m = 22		2.982322	3.514574	4.279707	5.475616	7.619951	12.699919										
m = 23		2.700629	3.109187	3.664081	4.461761	5.708542	7.944095	13.240159									
m = 24		2.484553	2.810813	3.236039	3.813570	4.643795	5.941443	8.268204	13.780340								
m = 25		2.313520	2.581941	2.920986	3.362877	3.963046	4.825811	6.174322	8.592281	14.320469							
m = 26		2.174753	2.400779	2.679319	3.031149	3.489705	4.112508	5.007812	6.407180	8.916331	14.860551						
m = 27		2.059891	2.253796	2.488030	2.776688	3.141303	3.616523	4.261959	5.189799	6.640021	9.240355	15.400592					
m = 28		1.963236	2.132138	2.332832	2.575273	2.874051	3.251450	3.743332	4.411400	5.371773	6.872845	9.564357	15.940596				
m = 29		1.880765	2.029768	2.204376	2.411860	2.662510	2.971407	3.361589	3.870134	4.560831	5.553736	7.105655	9.888339	16.480566			
m = 30		1.809559	1.942425	2.096288	2.276606	2.490882	2.749742	3.068757	3.471722	3.996928	4.710254	5.735688	7.338452	10.212303	17.020505		
m = 31		1.747447	1.867019	2.004072	2.162801	2.348830	2.569899	2.836968	3.166103	3.581850	4.123715	4.859669	5.917632	7.571237	10.536250	17.560417	

**Table A4:** The corresponding probabilities of MSE-RPs from P(IV)(0,1,0.1,5).

	P1	P2	P3	P4	P5	P6	P7	P8	P9	P10	P11	P12	P13	P14	P15	P16
m = 2	0.838195	0.161805														
m = 3	0.642074	0.317052	0.040874													
m = 4	0.480670	0.392115	0.113025	0.014190												
m = 5	0.360663	0.407760	0.177812	0.047784	0.005981											
m = 6	0.274208	0.389276	0.224062	0.086585	0.022992	0.002876										
m = 7	0.212063	0.354907	0.251141	0.122229	0.045963	0.012174	0.001523									
m = 8	0.166921	0.315534	0.262271	0.151081	0.070187	0.026203	0.006935	0.000868								
m = 9	0.133615	0.276854	0.261638	0.172141	0.092750	0.042471	0.015821	0.004186	0.000524							
m = 10	0.108618	0.241452	0.253125	0.185756	0.112147	0.059021	0.026891	0.010010	0.002649	0.000331						
m = 11	0.089537	0.210216	0.239839	0.192916	0.127760	0.074591	0.038905	0.017693	0.006584	0.001742	0.000218					
m = 12	0.074738	0.183188	0.224055	0.194845	0.139506	0.088447	0.050925	0.026464	0.012025	0.004474	0.001184	0.000148				
m = 13	0.063088	0.160034	0.207328	0.192766	0.147618	0.100223	0.062318	0.035661	0.018502	0.008404	0.003127	0.000827	0.000103			
m = 14	0.053791	0.140284	0.190660	0.187775	0.152501	0.109792	0.072685	0.044785	0.025555	0.013248	0.006017	0.002239	0.000592	0.000074		
m = 15	0.046280	0.123453	0.174657	0.180790	0.154634	0.117189	0.081801	0.053484	0.032807	0.018694	0.009688	0.004400	0.001637	0.000433	0.000054	
m = 16	0.040143	0.109092	0.159650	0.172542	0.154509	0.122550	0.089560	0.061522	0.039968	0.024460	0.013928	0.007216	0.003277	0.001219	0.000323	0.000040
m = 17	0.035076	0.096807	0.145794	0.163592	0.152594	0.126068	0.095944	0.068755	0.046827	0.030318	0.018532	0.010548	0.005465	0.002482	0.000923	0.000244
m = 18	0.030853	0.086264	0.133131	0.154354	0.149307	0.127969	0.100994	0.075103	0.053237	0.036087	0.023321	0.014245	0.008106	0.004200	0.001907	0.000709
m = 19	0.027304	0.077181	0.121636	0.145122	0.145009	0.128487	0.104790	0.080538	0.059101	0.041635	0.028147	0.018170	0.011095	0.006313	0.003270	0.001485
m = 20	0.024297	0.069324	0.111245	0.136102	0.140002	0.127850	0.107438	0.085064	0.064361	0.046864	0.032894	0.022203	0.014324	0.008744	0.004975	0.002577
m = 21	0.021732	0.062501	0.101876	0.127425	0.134531	0.126271	0.109056	0.088715	0.068988	0.051707	0.037474	0.026246	0.017699	0.011414	0.006967	0.003964
m = 22	0.019528	0.056550	0.093438	0.119175	0.128793	0.123942	0.109770	0.091541	0.072976	0.056121	0.041819	0.030220	0.021138	0.014246	0.009185	0.005606
m = 23	0.017623	0.051339	0.085843	0.111396	0.122939	0.121033	0.109703	0.093602	0.076335	0.060081	0.045882	0.034065	0.024573	0.017173	0.011570	0.007458
m = 24	0.015968	0.046758	0.079004	0.104106	0.117084	0.117689	0.108972	0.094969	0.079090	0.063578	0.049631	0.037732	0.027949	0.020138	0.014066	0.009474
m = 25	0.014521	0.042715	0.072840	0.097304	0.111316	0.114035	0.107689	0.095714	0.081273	0.066613	0.053045	0.041188	0.031222	0.023092	0.016626	0.011609
m = 26	0.013251	0.039135	0.067280	0.090980	0.105695	0.110173	0.105952	0.095908	0.082923	0.069196	0.056115	0.044407	0.034359	0.025995	0.019207	0.013822
m = 27	0.012130	0.035952	0.062257	0.085113	0.100266	0.106189	0.103853	0.095622	0.084081	0.071344	0.058836	0.047373	0.037332	0.028816	0.021773	0.016077
m = 28	0.011138	0.033114	0.057713	0.079678	0.095057	0.102148	0.101470	0.094922	0.084794	0.073080	0.061214	0.050076	0.040123	0.031528	0.024297	0.018343
m = 29	0.010255	0.030574	0.053595	0.074649	0.090085	0.098107	0.098871	0.093872	0.085106	0.074430	0.063257	0.052513	0.042719	0.034113	0.026753	0.020594
m = 30	0.009467	0.028295	0.049856	0.069998	0.085359	0.094107	0.096116	0.092527	0.085060	0.075419	0.064978	0.054684	0.045111	0.036555	0.029122	0.022807
m = 31	0.008761	0.026243	0.046457	0.065697	0.080880	0.090180	0.093254	0.090941	0.084700	0.076079	0.066391	0.056592	0.047295	0.038843	0.031389	0.024964
	P17	P18	P19	P20	P21	P22	P23	P24	P25	P26	P27	P28	P29	P30	P31	
m = 17	0.000031															
m = 18	0.000188	0.000023														
m = 19	0.000552	0.000146	0.000018													
m = 20	0.001170	0.000435	0.000115	0.000014												
m = 21	0.002053	0.000932	0.000347	0.000092	0.000011											
m = 22	0.003189	0.001652	0.000750	0.000279	0.000074	0.000009										
m = 23	0.004552	0.002590	0.001341	0.000609	0.000227	0.000060	0.000008									
m = 24	0.006107	0.003727	0.002120	0.001098	0.000499	0.000186	0.000049	0.000006								
m = 25	0.007818	0.005039	0.003075	0.001749	0.000906	0.000412	0.000153	0.000041	0.000005							
m = 26	0.009649	0.006497	0.004188	0.002556	0.001454	0.000753	0.000342	0.000127	0.000034	0.000004						
m = 27	0.011565	0.008072	0.005435	0.003503	0.002138	0.001216	0.000630	0.000286	0.000106	0.000028	0.000004					
m = 28	0.013537	0.009736	0.006795	0.004575	0.002949	0.001799	0.001024	0.000530	0.000241	0.000090	0.000024	0.000003				
m = 29	0.015538	0.011463	0.008244	0.005753	0.003873	0.002496	0.001523	0.000867	0.000449	0.000204	0.000076	0.000020	0.000003			
m = 30	0.017543	0.013230	0.009759	0.007017	0.004897	0.003297	0.002125	0.001297	0.000738	0.000382	0.000174	0.000065	0.000017	0.000002		
m = 31	0.019532	0.015015	0.011321	0.008349	0.006003	0.004189	0.002820	0.001818	0.001109	0.000631	0.000327	0.000148	0.000055	0.000015	0.000002	

## Appendix C. Information Gain and Related Covered Range

**Table A5:** The IG from different numbers of MSE-RPs of P(I)(1,5) and related covered range.

Number of MSE-RPs	IG	Covered Number of MSE-RPs	Covered Range
m = 6	90%≤IG≤95%	6	(1, 4.557853)
m = 7	90%≤IG≤95%	6	(1, 3.122798)
m = 8	90%≤IG≤95%	7	(1, 3.510284)
	95%≤IG≤98%	8	(1, 5.850473)
m = 9	90%≤IG≤95%	7	(1, 2.800594)
	95%≤IG≤98%	9	(1, 6.495583)
m = 10	90%≤IG≤95%	8	(1, 3.078516)
	95%≤IG≤98%	9	(1, 4.284110)
m = 11	90%≤IG≤95%	9	(1, 3.356274)
	95%≤IG≤98%	10	(1, 4.670642)
m = 12	90%≤IG≤95%	9	(1, 2.840237)
	95%≤IG≤98%	11	(1, 5.056998)
m = 13	90%≤IG≤95%	10	(1, 3.057155)
	95%≤IG≤98%	11	(1, 3.911437)
	98%≤IG≤99%	13	(1, 9.072026)
m = 14	90%≤IG≤95%	10	(1, 2.688677)
	95%≤IG≤98%	12	(1, 4.188889)
	98%≤IG≤99%	14	(1, 9.715537)
m = 15	90%≤IG≤95%	11	(1, 2.866721)
	95%≤IG≤98%	12	(1, 3.490814)
	98%≤IG≤99%	14	(1, 6.215339)
m = 16	90%≤IG≤95%	12	(1, 3.044731)
	95%≤IG≤98%	13	(1, 3.707577)
	98%≤IG≤99%	15	(1, 6.601282)
m = 17	90%≤IG≤95%	12	(1, 2.734661)
	95%≤IG≤98%	14	(1, 3.924305)
	98%≤IG≤99%	16	(1, 6.987163)
m = 18	90%≤IG≤95%	13	(1, 2.885668)
	95%≤IG≤98%	14	(1, 3.400669)
	98%≤IG≤99%	16	(1, 5.298153)
m = 19	90%≤IG≤95%	13	(1, 2.637633)
	95%≤IG≤98%	15	(1, 3.578605)
	98%≤IG≤99%	17	(1, 5.575373)
	99%≤IG	19	(1, 12.93129)
m = 20	90%≤IG≤95%	14	(1, 2.768771)
	95%≤IG≤98%	16	(1, 3.756524)
	98%≤IG≤99%	18	(1, 5.852566)
	99%≤IG	20	(1, 13.574203)

**Table A6:** The IG from different numbers of MSE-RPs of P(III)(0,1,0.2) and related covered range.

Number of MSE-RPs	IG	Covered Number of MSE-RPs	Covered Range
m = 7	90%≤IG≤95%	7	(0, 4.186923)
m = 8	90%≤IG≤95%	7	(0, 2.813247)
m = 9	90%≤IG≤95%	8	(0, 3.115306)
	95%≤IG≤98%	9	(0, 5.193419)
m = 10	90%≤IG≤95%	8	(0, 2.453194)
	95%≤IG≤98%	10	(0, 5.695577)
m = 11	90%≤IG≤95%	9	(0,2.670200)
	95%≤IG≤98%	10	(0,3.717980)
m = 12	90%≤IG≤95%	9	(0, 2.253536)
	95%≤IG≤98%	11	(0, 4.018854)
m = 13	90%≤IG≤95%	10	(0, 2.423314)
	95%≤IG≤98%	12	(0, 4.319518)
m = 14	90%≤IG≤95%	11	(0, 2.592731)
	95%≤IG≤98%	12	(0, 3.319267)
m = 15	90%≤IG≤95%	11	(0, 2.265638)
	95%≤IG≤98%	13	(0, 3.535249)
	98%≤IG≤99%	15	(0, 8.200847)
m = 16	90%≤IG≤95%	12	(0, 2.404934)
	95%≤IG≤98%	14	(0, 3.751119)
	98%≤IG≤99%	16	(0, 8.701244)
m = 17	90%≤IG≤95%	13	(0, 2.543988)
	95%≤IG≤98%	14	(0, 3.099726)
	98%≤IG≤99%	16	(0, 5.520828)
m = 18	90%≤IG≤95%	13	(0, 2.274345)
	95%≤IG≤98%	15	(0, 2.682864)
	98%≤IG≤99%	17	(0, 4.182610)
m = 19	90%≤IG≤95%	14	(0, 2.392483)
	95%≤IG≤98%	16	(0, 3.437139)
	98%≤IG≤99%	18	(0, 6.120974)
m = 20	90%≤IG≤95%	14	(0, 2.178159)
	95%≤IG≤98%	16	(0, 2.960250)
	98%≤IG≤99%	18	(0, 4.613867)
m = 21	90%≤IG≤95%	15	(0, 2.280909)
	95%≤IG≤98%	17	(0, 3.098813)
	98%≤IG≤99%	19	(0, 4.829431)
	99%≤IG	21	(0, 11.201569)

**Table A7:** The IG from different numbers of MSE-RPs of P(IV)(0,1,0.1,0.5) and related covered range.

Number of MSE-RPs	IG	Covered Number of MSE-RPs	Covered Range
m = 7	90%≤IG≤95%	7	(0, 4.574898)
m = 8	90%≤IG≤95%	7	(0, 3.071896)
m = 9	90%≤IG≤95%	8	(0, 3.398221)
	95%≤IG≤98%	9	(0, 5.663702)
m = 10	90%≤IG≤95%	8	(0, 2.676082)
	95%≤IG≤98%	10	(0, 6.206803)
m = 11	90%≤IG≤95%	9	(0, 2.909991)
	95%≤IG≤98%	10	(0, 4.049591)
m = 12	90%≤IG≤95%	10	(0, 3.143704)
	95%≤IG≤98%	11	(0, 4.374829)
m = 13	90%≤IG≤95%	10	(0, 2.639643)
	95%≤IG≤98%	12	(0, 4.699852)
m = 14	90%≤IG≤95%	11	(0, 2.822096)
	95%≤IG≤98%	12	(0, 3.610697)
m = 15	90%≤IG≤95%	11	(0, 2.467316)
	95%≤IG≤98%	13	(0, 3.844030)
	98%≤IG≤99%	15	(0, 8.915686)
m = 16	90%≤IG≤95%	12	(0, 2.617033)
	95%≤IG≤98%	14	(0, 4.077279)
	98%≤IG≤99%	16	(0, 9.456674)
m = 17	90%≤IG≤95%	13	(0, 2.766704)
	95%≤IG≤98%	14	(0, 3.369027)
	98%≤IG≤99%	16	(0, 5.998500)
m = 18	90%≤IG≤95%	13	(0, 2.474674)
	95%≤IG≤98%	15	(0, 3.551232)
	98%≤IG≤99%	17	(0, 6.322913)
m = 19	90%≤IG≤95%	14	(0, 2.601620)
	95%≤IG≤98%	16	(0, 3.733397)
	98%≤IG≤99%	18	(0, 6.647255)
m = 20	90%≤IG≤95%	15	(0, 2.728541)
	95%≤IG≤98%	16	(0, 3.215504)
	98%≤IG≤99%	18	(0, 5.009672)
m = 21	90%≤IG≤95%	15	(0, 2.480221)
	95%≤IG≤98%	17	(0, 3.365050)
	98%≤IG≤99%	19	(0, 5.242661)
m = 22	90%≤IG≤95%	16	(0, 2.590433)
	95%≤IG≤98%	18	(0, 3.514574)
	98%≤IG≤99%	20	(0, 5.475616)
	99%≤IG	22	(0, 12.699919)
